# The Type 2 Diabetes Factor Methylglyoxal Mediates Axon Initial Segment Shortening and Alters Neuronal Function at the Cellular and Network Levels

**DOI:** 10.1523/ENEURO.0201-21.2021

**Published:** 2021-10-06

**Authors:** Ryan B. Griggs, Duc V. M. Nguyen, Leonid M. Yermakov, Jeneane M. Jaber, Jennae N. Shelby, Josef K. Steinbrunner, John A. Miller, Carlos Gonzalez-Islas, Peter Wenner, Keiichiro Susuki

**Affiliations:** 1 Department of Neuroscience, Cell Biology, and Physiology, Boonshoft School of Medicine, Wright State University, Dayton, OH 45435; 2 Physiology Department, Emory University, School of Medicine, Atlanta, GA 30322; 3 Doctorado en Ciencias Biológicas, Universidad Autónoma de Tlaxcala, Tlax 90070, México

**Keywords:** axon initial segment, depolarization, methylglyoxal, multielectrode array, network activity, type 2 diabetes

## Abstract

Recent evidence suggests that alteration of axon initial segment (AIS) geometry (i.e., length or location along the axon) contributes to CNS dysfunction in neurological diseases. For example, AIS length is shorter in the prefrontal cortex of type 2 diabetic mice with cognitive impairment. To determine the key type 2 diabetes-related factor that produces AIS shortening we modified levels of insulin, glucose, or the reactive glucose metabolite methylglyoxal in cultures of dissociated cortices from male and female mice and quantified AIS geometry using immunofluorescent imaging of the AIS proteins AnkyrinG and βIV spectrin. Neither insulin nor glucose modification altered AIS length. Exposure to 100 but not 1 or 10 μm methylglyoxal for 24 h resulted in accumulation of the methylglyoxal-derived advanced glycation end-product hydroimidazolone and produced reversible AIS shortening without cell death. Methylglyoxal-evoked AIS shortening occurred in both excitatory and putative inhibitory neuron populations and in the presence of tetrodotoxin (TTX). In single-cell recordings resting membrane potential was depolarized at 0.5–3 h and returned to normal at 24 h. In multielectrode array (MEA) recordings methylglyoxal produced an immediate ∼300% increase in spiking and bursting rates that returned to normal within 2 min, followed by a ∼20% reduction of network activity at 0.5–3 h and restoration of activity to baseline levels at 24 h. AIS length was unchanged at 0.5–3 h despite the presence of depolarization and network activity reduction. Nevertheless, these results suggest that methylglyoxal could be a key mediator of AIS shortening and disruptor of neuronal function during type 2 diabetes.

## Significance Statement

Small changes in the structure of the axon initial segment (AIS) affect neuronal function and may be a key mediator of neurological complications in various disease states. However, the specific disease factors that mediate structural changes at the AIS are relatively unknown. This is the first study to show that increase of methylglyoxal is sufficient to reduce AIS length and modulate neuronal function at the cellular and network levels. Methylglyoxal is a disease factor implicated in a wide variety of conditions including type 2 diabetes, Alzheimer’s disease, and aging. Thus, these findings could significantly impact the understanding of neurological complications in several disease states and are of broad pathophysiological relevance.

## Introduction

The axon initial segment (AIS) is a specialized excitable domain within neurons that maintains neuronal polarity and regulates action potential generation (Rasband, 2010; Bender and Trussell, 2012). Voltage-gated sodium channels (Nav) are anchored at the AIS by sub-membranous cytoskeletal (e.g., βIV spectrin) and scaffolding (e.g., AnkyrinG) proteins (Nelson and Jenkins, 2017). Alteration of AIS constituents, such as mutations in ion channels or loss of protein complexes, is emerging as a key pathophysiology in a wide variety of neurological conditions in humans (for review, see Buffington and Rasband, 2011; Huang and Rasband, 2018). In the brains of animals, subtle changes in AIS geometry (i.e., length or location along the axon) are reported in models of type 2 diabetes (Yermakov et al., 2018), Alzheimer’s disease (Marin et al., 2016), aging (Atapour and Rosa, 2017; Ding et al., 2018), stroke (Hinman et al., 2013), multiple sclerosis-related demyelination (Hamada and Kole, 2015; Radecki et al., 2018), and neuropathic pain (Shiers et al., 2018). This suggests altered AIS geometry could be a shared mechanism of CNS dysfunction.

Patients with type 2 diabetes show slight decrement of cognitive performance across their lifespan and have increased risk of developing Alzheimer’s-like dementia (Biessels et al., 2014; Biessels and Despa, 2018). Some studies suggest that alteration of the AIS in brain regions important for cognition may change cognitive behavior in type 2 diabetes and related conditions. For example, concurrent cortical AIS shortening and cognitive impairment are present in type 2 diabetic db/db mice (Yermakov et al., 2018, 2019) and Alzheimer’s-like mice (Marin et al., 2016; Sri et al., 2019). In another study of mice, pharmacological treatment of neuropathic pain led to simultaneous reversal of cognitive impairment and AIS shortening in the infralimbic cortex (Shiers et al., 2018), supporting a link between AIS shortening in cortical neurons and cognition. To better understand the mechanisms underlying cognitive impairment in type 2 diabetes and related conditions, it is important to identify the factor(s) that initiate changes in AIS geometry in cortical neurons. In db/db mice, exercise treatment concomitantly ameliorated hyperglycemia and AIS shortening associated with type 2 diabetes (Yermakov et al., 2018), suggesting that type 2 diabetes-related factors such as high-glucose or insulin resistance may alter AIS geometry. Another potential mediator of AIS shortening in type 2 diabetes is the reactive carbonyl species and glucose metabolite methylglyoxal. We recently reported that methylglyoxal disrupts AnkryinG at paranodes in CNS myelinated axons (Griggs et al., 2018), suggesting that methylglyoxal may also affect the AnkyrinG-containing AIS in cortical neurons. Importantly, disruption of methylglyoxal metabolism, including pathophysiological increase of methylglyoxal or methylglyoxal-derived advanced glycation end-products, in humans is linked to the same neurological conditions as altered AIS geometry in animal models, including type 2 diabetes (Andersen et al., 2018), Alzheimer’s disease (Kuhla et al., 2007; Haddad et al., 2019), aging (Kuhla et al., 2006; Beeri et al., 2011; Srikanth et al., 2013), multiple sclerosis (Wetzels et al., 2019), and pain (Düll et al., 2019). The effect of methylglyoxal, glucose, or insulin on AIS geometry and the operation of neuronal networks is unknown.

To determine the key type 2 diabetes-related factor that leads to AIS shortening and test the hypothesis that this AIS shortening is associated with reduced neuronal activity at the cellular and network levels we used a reductionist approach. We exposed dense cultures of dissociated postnatal mouse cortex to modified levels of insulin, glucose, or methylglyoxal and quantified AIS geometry using AnkyrinG and βIV spectrin immunofluorescence. Since increase of methylglyoxal, but not insulin or glucose, was sufficient to shorten the AIS, we further characterized the effect of methylglyoxal on our cultures by assessing cell viability, the reversibility of AIS shortening, and the cell-type specificity of AIS shortening using the excitatory neuron marker Ca^2+^/calmodulin-dependent protein kinase IIα (CaMKIIα). Moreover, we evaluated the temporal relationship between methylglyoxal-evoked changes in AIS length, single-cell excitability, and neuronal network activity. We are the first to uncover that short-term elevation of methylglyoxal, a metabolic factor whose pathophysiological increase is implicated in many neurological disease states including type 2 diabetes, changes AIS geometry and transiently alters neuronal function at both the single-cell and network level.

## Materials and Methods

### Animals

Adult male and female C57BL/6J mice (The Jackson Laboratory; RRID:IMSR_JAX:000664) aged 7–30 weeks were used for in-house breeding for postnatal cortical cultures. Mice were housed in Laboratory Animal Resources at Wright State University at 21–25°C and 20–60% humidity under 12/12 h light/dark conditions (lights on 7 A.M. to 7 P.M.) with *ad libitum* access to food and water. Mice were provided wood-chip bedding, toilet paper roll cardboard tubes, shredded accordion paper, and thick cotton pads for enrichment and group housed except when single housing was required for breeding purposes. All animal procedures were approved by the Institutional Animal Care and Use Committee at Wright State University (Animal Use Protocol #1113 and #1190) and conducted in accordance with the National Institutes of Health Office of Laboratory Animal Welfare Guide for the Care and Use of Laboratory Animals and the ARRIVE 2.0 guidelines (Percie du Sert et al., 2020).

### Postnatal cortical cultures

Preparation and culturing of dissociated cells from postnatal mouse cortices was similar to as described previously (Beaudoin et al., 2012) using the following materials: Neurobasal-A Medium (10888-022, Thermo Fisher Scientific), Neurobasal Plus Medium (A3582901, Thermo), Neurobasal-A without D-glucose or sodium pyruvate (A2477501, Thermo), B-27 Supplement (17504-044, Thermo), B-27 Plus Supplement (A3582801, Thermo), B-27 without insulin (A1895601, Thermo), GlutaMAX Supplement (35050061, Thermo), sodium pyruvate (11360070, Thermo), Hibernate-A Medium (A1247501, Thermo), gentamycin (G1272, Sigma-Aldrich), papain (PDS kit: LK003178, Worthington Biochemical Corporation), DNase I (90083, Thermo), fetal bovine serum (FBS; 26140-087, Thermo), poly-L-lysine (P2636, Sigma), coverslips precoated with poly-L-lysine (12 mm #1.5 Biocoat 354085, Corning; or GG-12-1.5-PLL, Neuvitro Corporation), Biosafety Cabinet Class II A2 (NU-540-500, Nuaire), CO_2_ incubators (NU-5710, Nuaire).

#### Preparation of dissociated cortical cells

Plating media (Neurobasal-A or Neurobasal Plus, 2 mm GlutaMAX, 2% B-27 or 2% B-27 Plus, 5% FBS, 10 μg/ml gentamycin) and growth media (Neurobasal-A or Neurobasal Plus, 2 mm GlutaMAX, 2% B-27 or 2% B-27 Plus) were prepared fresh, stored at 4°C, and used within 7 d. Papain (40 U/ml) was solubilized in Neurobasal-A or Hibernate-A Medium containing DNase I (40 U/ml) and prewarmed at 37°C for 20 min. Whole brains were removed from decapitated male and female mouse pups at postnatal day (P)0 and covered in ice-cold Hibernate-A. The left and right cortices were separated, meninges removed, minced into ∼2-mm^3^ pieces and transferred to a sterile 14-ml culture test tube containing 1-ml ice-cold Hibernate-A. Cells were enzymatically and mechanically dissociated by adding 1 ml of prewarmed, activated papain/DNase solution (20 U/ml final concentration), incubating in a 37°C bead bath for 20 min with gentle swirling every 5 min, followed by gentle trituration three to five times using a fire-polished 9-inch Pasteur pipet. Triturated solution containing cells and tissue debris was passed through a sterile 100-μm strainer, transferred to a 15-ml polystyrene centrifuge tube, centrifuged 5 min at 250 × *g*, supernatant decanted, cell pellet resuspended in ∼1 to 2-ml plating media per cortical pair, cells counted using a hemocytometer (0267110, Thermo), and concentration adjusted to 1 × 10^6^ cells/ml with additional plating media. Preparation of dissociated cells from both cortices from one postnatal mouse pup brain typically yielded circa 2 × 10^6^ cells; however, sometimes cells dissociated from multiple pup brains were combined into one tube if larger numbers of cells were needed for a given experiment, and this was deemed as one culture preparation, or simply one culture. For whole-cell patch clamp electrophysiology, cultures were prepared as above but from embryonic day (E)18 cortices (C57ECX, BrainBits LLC) using Neurobasal Medium (21103049, Thermo) and Hibernate-E (A1247601, Thermo).

#### Plating

Coverslips (12 mm), tissue culture dishes (35 mm), and multielectrode arrays (MEAs) were precoated with poly-L-lysine diluted to 0.1 mg/ml in 0.1 m borate buffer pH 8.5 and incubated >1 h to overnight at 37°C, washed with sterile water 2 times, allowed to completely dry in biosafety cabinet, and stored for up to two weeks at 4°C until use. Dissociated cortical cells from P0 pups were pipetted onto precoated: 12-mm coverslips contained in 35-mm dishes (100,000 cells in 100 μl per coverslip, three to four coverslips per 35-mm dish) for immunofluorescence and cell viability assays, 35-mm dishes (1,000,000 cells in 1 ml) for Western blotting, or MEAs (50,000 cells in 50 μl) for neuronal network activity recordings, allowed to settle onto the substrate for at least 10 min, and plating media added up to a final volume of 2 ml (35-mm dishes) or 1 ml (MEAs). After overnight incubation, all the plating media was exchanged for growth media and cultures were maintained in growth media with 50% media changes every 3–4 d for coverslips/dishes or up to 7 d for MEAs where specialized membrane lids were used to prevent evaporation (ALAMEA-MEM5, ALA Scientific). For whole-cell patch clamp electrophysiology 50,000 dissociated cortical cells from E18 pups were plated on 22-mm coverslips (C022001, Matsunami Glass USA Inc.) precoated with FBS, poly-L-lysine, and laminin at a similar cell density to the 12-mm coverslips used for immunofluorescence analyses.

#### Characterization

Previous studies indicate that the AIS matures during the first week of culture and maximal levels of both AnkyrinG (Galiano et al., 2012) and Nav (Yang et al., 2007) protein are present circa day *in vitro* (DIV)7. Therefore, drug exposure experiments were initiated on or after DIV8. The percentage of microtubule associated protein 2 (MAP2) positive neurons MAP2+ (78.2 ± 2.5%) and glial fibrillary acidic protein (GFAP) positive astrocytes (21.8 ± 2.5%) at DIV8 was similar to the percentage of MAP2+ (72.6 ± 3.4%) and GFAP+ (27.4 ± 3.4%) cells at DIV14 (*n* = 12 coverslips from four cultures). This distribution of astrocytes (∼20%) is greater than the 6–8% previously reported in low density cortical cultures prepared from postnatal P0 to P1 (Beaudoin et al., 2012) or embryonic E17 (Bélanger et al., 2011) mice. The percentage of MAP2+ neurons that were CaMKIIα+ (78.5 ± 1.07%, *n* = 12 coverslips from three cultures) was greater than CaMKIIα− (21.5 ± 1.07%, *n* = 12 coverslips from three cultures) at DIV14. This distribution of putative inhibitory cells (∼20%) agrees with previous mouse studies of GABA+ cells in cortical cultures (Beaudoin et al., 2012) or GAD67+ cells in hippocampal cultures (Prestigio et al., 2019). The density of cells in our cortical cultures (empirically determined to be ∼1400 cells/mm^2^) is about three times the density of a previous study of AIS plasticity in hippocampal cultures where 45,000 cells were seeded onto 13-mm coverslips (calculated density of ∼440 cells/mm^2^; Evans et al., 2013). This is notable, since a previous study in rat embryonic hippocampal cultures indicates that cell density alters neuronal excitability and AIS geometry (Guo et al., 2017).

### Drug exposure

Dissociated P0 cortical cells were cultured on coverslips contained in 35-mm dishes for immunofluorescence or directly in 35-mm dishes for Western blotting. Cultures were exposed to insulin (A11382II, Thermo), D-(+)-glucose (G8270, Sigma), D-mannitol (M4125, Sigma), methylglyoxal (6.49 m stock, M0252, Sigma), or tetrodotoxin (TTX; T-550; Alomone Labs) by dilution of the drug in growth media to 2× final concentration, syringe-filtration through 0.22-μm PES membranes (229746, CELLTREAT Scientific Products), prewarming to 37°C, and then adding 1 ml to 35-mm dishes containing 1 ml of conditioned medium for a 1× final concentration.

#### Insulin exposure

Insulin resistance was generated in our postnatal cortical cultures as previously described in embryonic mouse cortical cultures (Kim et al., 2011) with modification. Cultures were grown in growth media containing standard Neurobasal-A from DIV0 to DIV10. Since the concentration of insulin in standard Neurobasal-A media is already supraphysiological (∼700 nm), we generated insulin resistance by first omitting insulin completely for 24 h at DIV10–DIV11 followed by 24 h of exposure at DIV11–DIV12 to standard growth media containing ∼700 nm insulin or growth media modified to contain 0, 20, or 100 nm insulin. We validated the generation of insulin resistance by challenging a subset of cultures grown on 35-mm dishes with 20 nm insulin for 15 min followed by quantification of the phosphorylated to non-phosphorylated Akt ratio (pAkt/Akt) using Western immunoblotting band density. Cultures used to quantify AIS length did not undergo the 15-min insulin challenge.

#### Glucose exposure

To model hyperglycemia in mature (DIV10–DIV11) cultures, coverslips were grown in standard culture conditions from DIV0 to DIV10 and then exposed to unmodified media containing 25 mm glucose or media modified to contain 50 mm high glucose or 25 mm glucose plus 25 mm mannitol. Mannitol was added to control for potential effect of hyperosmolality generated by doubling the amount of glucose in standard culture conditions (25 mm) to 50 mm high glucose.

#### Methylglyoxal exposure

Cultures were grown in standard growth media followed by methylglyoxal exposure initiated on DIV13 for Western immunoblotting and MEA analyses, DIV8–DIV13 for immunofluorescence quantitation of AIS geometry or viability, and DIV12–DIV14 for whole-cell electrophysiology.

### Western blotting of postnatal cortical cultures grown on 35-mm dishes

Dissociated cells from P0 mice cortices were grown on poly-L-lysine coated 35-mm tissue culture treated dishes as described above. Protein lysates for Western immunoblotting were prepared by rinsing dishes in ice-cold PBS twice then adding ice-cold lysis buffer (25 mm Tris HCl at pH 7.5, 150 mm NaCl, 1% Triton X-100, 0.5% deoxycholate, 0.1% SDS, and 10 mm EDTA) containing protease inhibitor cocktail (P8340, Sigma) and phosphatase inhibitor cocktail (78428, Thermo). Cells were collected using a sterile cell scraper (08-100-241, Fisher) in 2 × 100 μl volumes of ice-cold lysis buffer, transferred to ice-cold 1.5-ml tubes, vortexed, incubated on ice for 10 min, then centrifuged at 18,000 × *g* for 10 min at 4°C in a Sorvall Legend Micro 21R centrifuge (Thermo). After centrifugation, supernatant was collected into fresh, ice-cold tubes and protein concentrations were measured using a Pierce BCA Protein Assay (232525, Thermo) or Coomassie Plus Bradford Assay (23236, Thermo). Samples (10-μg protein) were denatured at 95°C for 5 min in 4× Laemmli sample buffer (1610747, Bio-Rad) or Novex NuPAGE LDS sample buffer (NP0007, Thermo) with β-mercaptoethanol (1610710, Bio-Rad) or 10× reducing agent (DTT; B0009, Thermo), then run on either a 4–20% Mini-PROTEAN TGX stain-free gel (4568096, Bio-Rad) or on 4–12% gels using the Novex Bolt mini-gel system (NW0412B, B1000B, BT000614, Thermo). The gel was then transferred to nitrocellulose membrane with 0.45 μm pore size (1620115, Bio-Rad). Membranes were blocked for 1 h in Pierce protein-free (PBS) blocking buffer (37572, Thermo) for MG-H1 or 20 mm Tris, pH 8.0 and 0.05% (v/v) Tween 20 (TBST) containing 4% (w/v) milk for all other antigens. Membranes were incubated overnight at 4°C in primary antibody diluted (1:1000) in TBST with milk or protein-free blocking buffer. Primary antibody was washed 3 × 5 min in TBST followed by incubation of the membrane in horseradish peroxidase (HRP)-conjugated secondary antibody (1:10,000 or 1:20,000; Jackson ImmunoResearch) for 1 h at room temperature. Signals generated by Pierce ECL Plus Western Blotting Substrate (32132, Thermo) were detected using a ChemiDoc MP Imaging System (Bio-Rad) or Azure 600 (Azure Biosystems). Quantification of the band density was performed using ImageLab software (Bio-Rad). The densities of the bands of interest were normalized to the relative expression of GAPDH or Total Protein staining by TGX stain-free precast gels (4568096, Bio-Rad), SYPRO Ruby (S4942, Sigma), or Azure Ponceau (10147-344, VWR).

### Immunofluorescent analysis of postnatal cortical cultures grown on 12-mm coverslips

Immunostaining of postnatal cortical cultures was performed as follows. Custom humidity chambers were created by super gluing caps from 1.5-ml microcentrifuge tubes to the bottom of 100-mm Petri dishes and adding a piece of Kimwipe wetted with dH_2_O. After drug exposure, media was removed and dishes were rinsed three times in prewarmed 1× PBS (137 mm NaCl, 2.7 mm KCl, and 11.9 mm phosphate) then fixed in room temperature 4% paraformaldehyde (PBS, pH 7.4) for 20 min. After washing 3 × 5 min in PBS, coverslips were taken from the 35-mm dishes and dabbed on a Kimwipe to remove liquid then placed onto the microcentrifuge caps within the humidity chambers to complete the immunostaining process. Coverslips were blocked in PBSTGS (1× PBS, 0.1% Triton X-100, 10% goat serum) for 30–60 min. Fifty microliters of primary antibody diluted in PBSTGS was added to each coverslip and then coverslips within the custom humidity chambers were incubated in primary antibody overnight at 4°C. After 3 × 5 min wash in PBSTGS, appropriate secondary antibodies were diluted in PBSTGS, added to the coverslips, and incubated in the dark at room temperature for 1 h. Coverslips were washed for 5 min each in PBSTGS, 0.01 m phosphate buffer, 0.005 m phosphate buffer, allowed to air dry, then mounted on non-coated slides using either KPL Mounting Medium (71-00-16, VWR) or ProLong Gold Antifade Mountant without DAPI (36934, Thermo) or with DAPI (P36941, Thermo). In some instances, Hoechst was added during the PBSTGS wash step after secondary antibody incubation. After mounting onto slides, coverslips were secured using clear nail polish and stored at −20°C until imaging analysis.

The primary antibodies used are listed in Table 1. AffiniPure minimally cross-reactive secondary antibodies used for immunofluorescent imaging were conjugated to Alexa Fluor (594, 488, 350) or AMCA (Jackson ImmunoResearch). Secondary antibodies used for western immunoblotting were conjugated to HRP (Jackson ImmunoResearch). Hoechst or DAPI were used to stain cell nuclei.

**Table 1:** Primary antibodies used for immunofluorescent imaging or Western immunoblotting

Antigen	Dilution	Species	Isotype	Manufacturer information and RRID citation
Akt	1:1000	Rabbit	IgG	Cell Signaling Technology catalog #9272, RRID:AB_329827
Phospho-Akt (Ser473)	1:1000	Rabbit	IgG	Cell Signaling Technology catalog #9271, RRID:AB_329825
AnkyrinG	1:400	Mouse	IgG2a	UC Davis/NIH NeuroMab Facility catalog #75-146, RRID:AB_10673030
βIV spectrin	1:400	Rabbit	IgG	M. N. Rasband, Baylor College of Medicine, TX, catalog #bIV SD, RRID:AB_2315634
CaMKIIα	1:400	Mouse	IgG1	Thermo Fisher Scientific catalog #MA1-048, RRID:AB_325403
GAPDH	1:1000	Mouse	IgG1	Enzo Life Sciences catalog #ADI-CSA-335-E, RRID:AB_2039148
GFAP	1:1000	Mouse	IgG1	UC Davis/NIH NeuroMab Facility catalog #75-240, RRID:AB_10672299
MAP2	1:1000	Chicken	IgY	EnCor Biotechnology catalog #CPCA-MAP2, RRID:AB_2138173
MG-H1	1:1000	Rat	IgG	T. Fleming and P. Nawroth, Heidelberg University, Germany; 1D2 tissue culture supernatant

Images of immunostained coverslips were obtained using a fluorescence microscope (Axio Observer Z1 with Apotome 2 fitted with AxioCam Mrm CCD camera, Carl Zeiss Microscopy LLC) and measurement of AIS length was performed using the curve spline measurement tool in ZEN2.0 (Carl Zeiss) or a MATLAB script adapted from previous studies (Grubb and Burrone, 2010; Yau et al., 2014). Our comparison of AIS length measured by eye versus by custom MATLAB script yielded similar results, which is consistent with a strong correlation (Spearman *r* > 0.8) of AIS length measured by eye with AIS measurement using custom-written MATLAB scripts as reported previously (Grubb and Burrone, 2010; Evans et al., 2015; Guo et al., 2017; Fruscione et al., 2018; Zhang et al., 2019). Measurement of AIS start location (distance from the edge of the soma to the start of the AIS) was performed as described previously (Guo et al., 2017). Only continuous, non-overlapping AISs originating from a MAP2+ cell were quantified by an observer blinded to experimental treatment.

### Cell viability assay

Cell viability was determined using a commercially available dual-wavelength fluorescent kit (LIVE/DEAD viability/cytotoxicity assay kit: L3224, Invitrogen). Live cells are detected by intracellular esterase activity using the dye calcein-AM (∼495/515 nm ex/em; green). Dead cells are detected by a large increase in fluorescence of the dye ethidium homodimer-1 when bound to nucleic acids, which only occurs if the plasma membrane is compromised (∼495/635 nm ex/em; red). Optimal dilution (1:2000 for each dye) of calcein-AM (4 mm in DMSO) and EthD-1 (2 mm in DMSO:H_2_O 1:4) stocks was empirically determined by comparing signal:noise ratios in the green and red channels. We validated the live/dead assay by exposing mature cortical cultures to either 1× PBS or 100% methanol for 15 min and confirming methanol-induced cell death. After drug exposure, coverslips were rinsed 3× in 1× PBS and incubated in 80 μl of calcein-AM/EthD-1/PBS solution in the dark at room temperature for 15 min. Excess solution was removed and coverslips were mounted in 5 μl calcein-AM/EthD-1/PBS solution, sealed using clear nail polish to minimize evaporation, and immediately imaged to avoid signal decay of calcein-AM fluorescence. The number of cells displaying green or red fluorescence was quantified and data are presented as percentage of number of live cells counted divided by total cells counted (number of live cells + number of dead cells) normalized to media control.

### Whole-cell patch clamp recordings

Patch clamp tight seals (1–3 GΩ) were obtained under voltage clamp using thin-walled borosilicate glass capillary (World Precision Instruments) electrodes pulled in two stages using a P-87 Flaming/Brown Micropipette Puller (Sutter Instruments) to obtain microelectrodes with resistances between 6 and 7 MΩ. Once whole-cell configuration was achieved, voltage clamp was maintained at 70 mV for a period of 5 min to allow stabilization before switching to current clamp configuration to record resting membrane potential. Voltage signals were acquired with an Axopatch 200B patch clamp amplifier (Molecular Devices), digitized (Digidata 1200, Molecular Devices), and recorded using Axon pCLAMP 10 (Molecular Devices). Recordings were terminated if a 20% increase in input resistance was observed. Current clamp recordings were filtered online at 10 kHz and digitized at 20 kHz. A liquid junction potential of −9 mV was experimentally measured and corrected for (Neher, 1992). The patch pipette solution contained the following: 5 mm NaCl, 100 mm K-gluconate, 36 mm KCl, 10 mm HEPES, 1.1 mm EGTA, 1 mm MgCl_2_, 0.1 mm CaCl_2_, 1 mm Na_2_ATP, and 0.1 mm MgGTP with osmolarity of 280–300 mOsm and pH adjusted to 7.3 with KOH. Recordings were obtained in artificial CSF (aCSF) containing the following: 126 mm NaCl, 3 mm KCl, 2 mm CaCl_2_, 1.5 mm MgSO_4_, 1 mm NaH_2_PO_4_, 25 mm HEPES, and 25 mm D-glucose with pH adjusted to 7.4 using NaOH. Drug exposure was performed by infusing control aCSF or aCSF containing drug solution at a similar rate of 3 ml/min. Action potential thresholds were assessed using a current injection step protocol (500-ms duration per step; 20 steps; 10-pA increments; 0–200 pA). A 200-ms hyperpolarizing current of 20 pA was delivered 800 ms before every step pulse to determine input resistance and serve as an indicator of the reliability of the step. The current injection step protocol was repeated three times for each cell, and the average value was used for analysis. Rheobase current, threshold voltage and membrane resistance measurements were conducted offline using Clampfit software (Molecular Device). Analysis was performed blinded to experimental treatment.

### MEA recording of neuronal network activity

MEA recordings were performed as previously described (Wagenaar et al., 2006; Hales et al., 2010; Fong et al., 2015) using the MEA2100-60-System (Multi Channel Systems) that includes MEA2100-HS60 headstage with integrated heating element, MCS-interface board 3.0 multiboot, Multi Channel Suite acquisition and analysis software, and temperature controller. MEAs (60MEA200/30iR-Ti-g, Multi-Channel Systems) were precoated with poly-L-lysine as described above, a 25-μl drop of 40 μg/ml laminin (L2020, Sigma) was carefully pipetted directly onto the center of the electrode array, and MEAs were incubated at 37°C during preparation of postnatal cortical cells. Before plating the cells, laminin was incompletely aspirated leaving a wetted area that localizes dispersion of the cell suspension on top of the electrode array. MEAs were maintained by 50% media changes every 3–7 d with fresh prewarmed growth media using specialized gas-permeable membrane ALA lids (ALAMEA-MEM5, ALA Scientific Instruments) to prevent evaporation that can result in increased tonicity and toxicity (Potter and DeMarse, 2001). MEAs remained in the incubator during the experiment except during the 15 min recording period at each time point. Recordings were performed with the MEA2100-60-System on the benchtop in ambient air with headstage heated to 37°C. The ALA lids prevented contamination during repeated recordings across several days. Since we observed that 50% media changes altered spiking activity within the first several hours, experimental recordings were performed the day after media changes. Vehicle control (2-μl growth media) or methylglyoxal (2 μl of 500× final concentration) were added to MEAs containing 1-ml conditioned media resulting in a 1× final concentration. The pH was similar before and after addition of either media or methylglyoxal. Recordings were digitized at 20,000 Hz. Threshold for spike detection was set at mean noise ± 5 SDs on a per channel basis as previously reported (Charkhkar et al., 2015). Multi Channel Suite (Multi Channel Systems) and Neuroexplorer 5.0 (Nex Technologies) were used to create raster plots and extract network spiking and bursting parameters.

MEA-wide network spike frequency was obtained by dividing the recording duration (15 min) into the total amount of spikes from 59 channels (reference channel was omitted; Wagenaar et al., 2006; Fong et al., 2015). Network bursts were extracted using the MCS Analyzer Burst Analyzer tool with settings of 50-ms maximum interval to start/end burst, 100-ms minimum interval between bursts, 50-ms minimum duration of burst, at least five spikes within a burst, and at least 15 channels participating in the network burst. MEA-wide network bursting, defined as synchronized spiking activity across all or most of the recording channels, was consistently seen in all MEA preparations. The variance of network spike frequency in the MEAs at baseline was relatively large; therefore, results are presented as percentage of baseline network spike frequency within each MEA. To account for heterogeneity of network function between cultures as previously reported (Wagenaar et al., 2006), we used replicate (sister cultures) MEAs from multiple cultures.

### Experimental design and statistical analysis

Statistics for both n=AIS/image/coverslip and n=culture preparation (average of AIS geometry, cell viability, or cell distribution outcomes within a single culture preparation) are reported in the results text. Comparison of the means between two groups was performed using unpaired, homoscedastic *t* test. Effect of insulin, glucose, or methylglyoxal concentration was analyzed using one-way ANOVA and/or linear regression. An interaction of time and drug was determined using two-way ANOVA. Multiple comparison *post hoc* tests are listed in the results text. An α value of α = 0.05 was used to determine statistical significance. All data were analyzed using Prism 9.1 (GraphPad) or SAS 9.4 (SAS Institute). Data for n=AIS are presented as cumulative frequency distributions or scatter plots where the width of the distribution of data points is proportional to the number of data points at a given Y value. Data for n=culture preparation (larger open symbols) are overlayed on the scatter plots showing each AIS, image, or coverslip (small gray dots). Figures show mean ± SEM and the *p* value or “ns” (not significant) for n=culture preparation (immunofluorescence) or n=MEA/cell (electrophysiology).

## Results

### Insulin sensitivity manipulation does not affect AIS length

**Figure 1.**
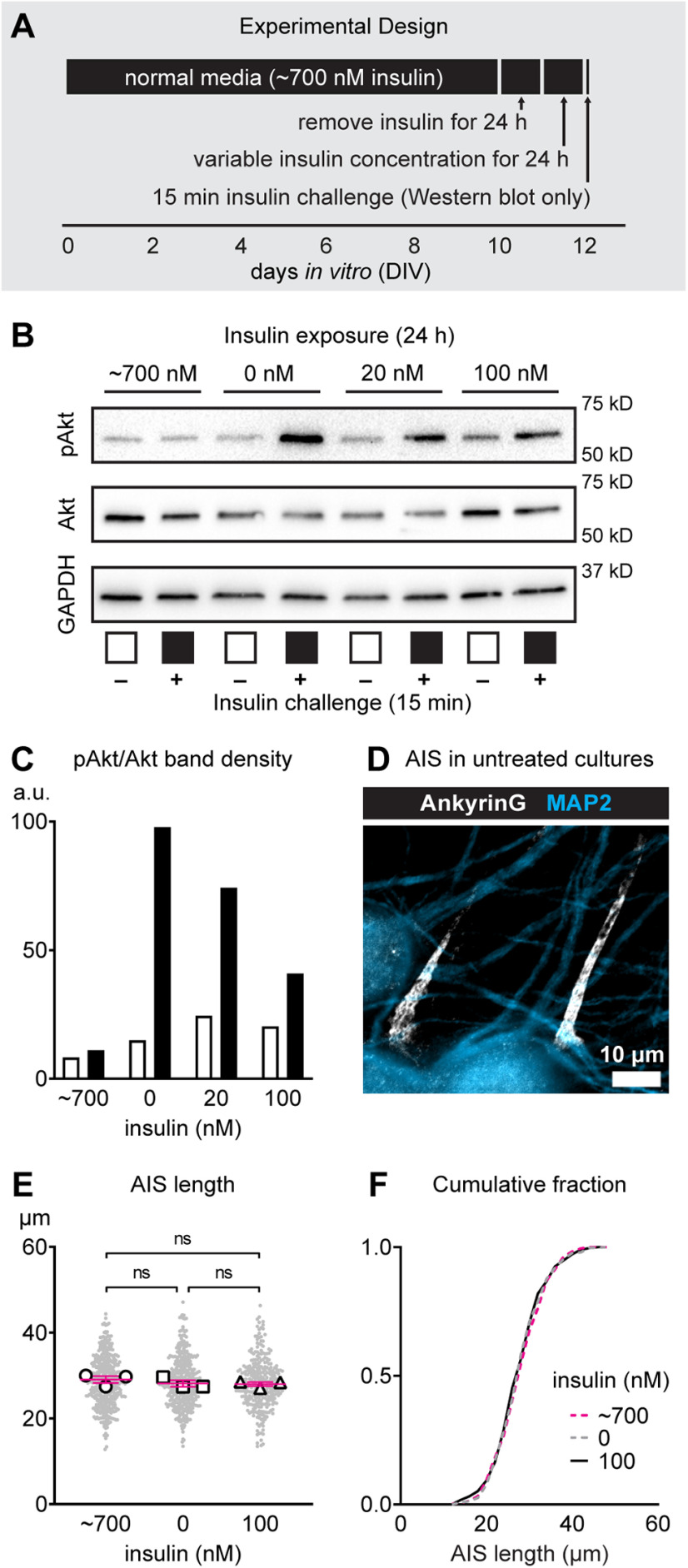
Effect of altering insulin on AIS length in mouse cortical cultures. ***A***, Schematic showing the experimental design and timeline of variable insulin exposure to induce insulin resistance (revealed by Western blotting after a 15-min challenge to insulin) in culture. ***B***, ***C***, Western immunoblotting results showing phosphorylation status of Akt after inducing acute insulin resistance. White bars denote absence of 15-min insulin challenge and black bars denote presence of 15-min insulin challenge that was used to reveal insulin sensitivity status. a.u. (arbitary units). ***D***, Representative image of mature postnatal cortical cultures at DIV10 showing MAP2 (somatodendritic domain) and AnkyrinG (AIS). Scale bar: 10 μm. ***E***, AIS length after inducing conditions of high insulin resistance (normal media containing ∼700 nm insulin), no insulin resistance (0 nm insulin), and moderate insulin resistance (100 nm insulin). Small gray dots indicate each AIS and larger open symbols represent the average AIS length for each culture preparation (293–416 AIS were analyzed from *n* = 3 cultures). ns (not significant) indicates statistical comparison at the culture preparation level. ***F***, Cumulative fractional distribution of AIS lengths after 24 h of exposure to ∼700, 0, or 100 nm insulin.

Type 2 diabetes is canonically characterized by insulin resistance and elevated glucose. First, we tested whether altering insulin sensitivity in postnatal cortical cultures affects AIS length. We used a protocol previously established to produce insulin resistance in embryonic mouse primary cortical cultures (Kim et al., 2011). As shown in the experimental design (Fig. 1*A*), we removed insulin for 24 h in mature cultures, varied the insulin concentration for an additional 24 h, then validated the presence of insulin resistance via Akt immunoblotting and quantified AIS length by immunostaining for AnkryinG, the master organizer of the AIS (Rasband, 2010). Previous studies suggest that a 24 h period of time might be sufficient to observe changes in AIS geometry in cultured CNS neurons (Grubb and Burrone, 2010; Chand et al., 2015). Quantification of phosphorylated Akt and total Akt immunoblot band density revealed that increasing the concentration of insulin for 24 h led to a lower pAkt/Akt ratio when challenged with insulin for 15 min (Fig. 1*B*,*C*). Decreased phosphorylation of Akt is indicative of insulin resistance. A representative image of the AIS (AnkyrinG) and the somatodendritic domain (MAP2) in untreated mature cultures is shown (Fig. 1*D*). AIS length was similar after 24 h of exposure to 700 nm (28.61 ± 0.28 μm, *n* = 416 AIS from three cultures), 0 nm (28.41 ± 0.29 μm, *n* = 396 AIS from three cultures), or 100 nm (28.20 ± 0.34 μm, *n* = 293 AIS from three cultures) insulin (insulin concentration: *F*_(2,1102)_ = 0.45, *p *=* *0.64; Tukey) when n=AIS. When analyzed at the culture preparation level, AIS length was also similar (insulin concentration: *F*_(2,6)_ = 0.68, *p *=* *0.54; Tukey; *n* = 3 cultures; Fig. 1*E*). Cumulative frequency plot shows overlapping distributions of AIS lengths after variable insulin exposure (Fig. 1*F*), indicating that no changes in AIS length are observed in the entire population of cortical neurons.

### Glucose does not appreciably change AIS length

**Figure 2.**
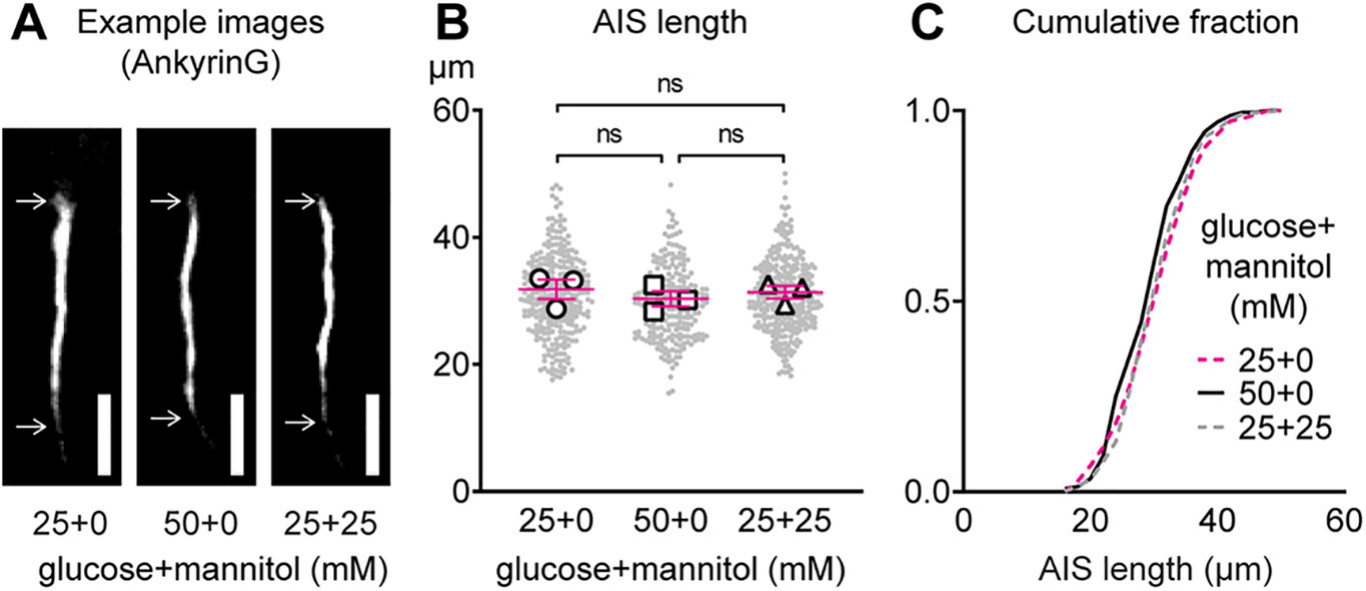
Effect of altering glucose on AIS length in mouse cortical cultures. ***A***, Example images of the AIS after 24 h of exposure to normal culture media containing 25 mm glucose, high-glucose media containing 50 mm glucose, or normal media containing 25 mm glucose with 25 mm mannitol added as an osmotic control in mature cultures (DIV10–DIV11). Scale bar: 10 μm. ***B***, Quantification of AIS length after 24 h of exposure to 25, 50, or 25 mm glucose plus 25 mm mannitol in mature cultures (DIV10–DIV11). Small gray dots indicate each AIS and larger open symbols represent the average AIS length for each culture preparation (236–320 AIS were analyzed from *n* = 3 cultures). ns (not significant) indicates statistical comparison at the culture preparation level. ***C***, Cumulative fractional distribution of AIS lengths after 24 h of exposure to 25, 50, or 25 mm glucose plus 25 mm mannitol.

Next, we tested whether elevating glucose levels in mature postnatal cortical cultures changes AIS length. Example images are shown 24 h after exposing mature cortical cultures to normal Neurobasal-A growth media containing 25 mm glucose, high-glucose media containing 50 mm glucose, or normal media containing 25 mm glucose with 25 mm mannitol added to control for the elevated osmolarity in high-glucose media (Fig. 2*A*). When analyzed at the AIS level, AIS length was significantly altered (main effect of treatment: *F*_(2,844)_ = 3.56, *p *=* *0.03; Tukey) after 24 h of exposure to 25 mm glucose (30.92 ± 0.37 μm, *n* = 291 AIS from three cultures), 50 mm high glucose (29.66 ± 0.37 μm, *n* = 236 AIS from three cultures), or 25 mm glucose plus 25 mm mannitol (30.81 ± 0.32 μm, *n* = 320 AIS from three cultures). However, when analyzed at the culture preparation level there was no difference in AIS length (main effect of treatment: *F*_(2,6)_ = 0.37, *p *=* *0.70; Tukey; *n* = 3 cultures; Fig. 2*B*). Cumulative frequency distributions show similar AIS length after altering the concentration of glucose or mannitol (Fig. 2*C*). Taken together, these results show that altered insulin sensitivity (Fig. 1) and high glucose (Fig. 2) do not appreciably change AIS length after 24 h of exposure in our cortical cultures.

### Methylglyoxal exposure increases cellular methylglyoxal-derived hydroimidazolone (MG-H1)

**Figure 3.**
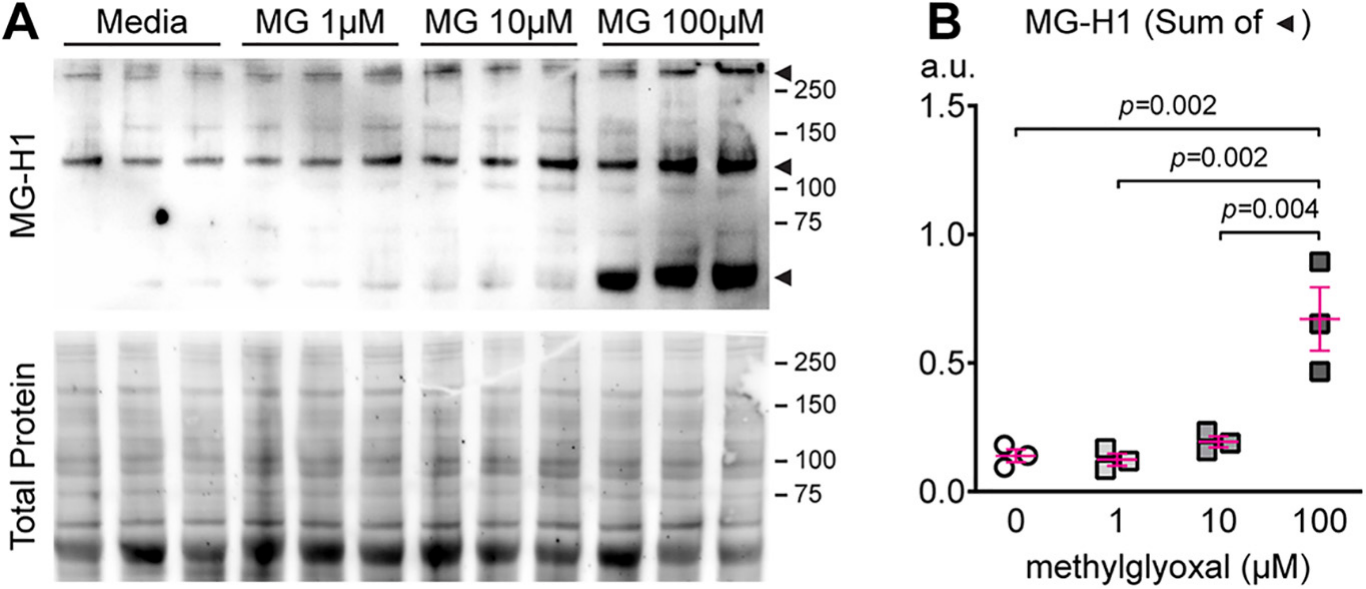
Methylglyoxal increases cellular methylglyoxal-derived hydroimidazolone (MG-H1) in mouse cortical cultures. ***A***, Western immunoblot image of cultures exposed to methylglyoxal (0–100 μm; 24 h; *n* = 3 cultures) showing MG-H1 (unpurified rat anti-MG-H1 clone 1D2 antibody) and Total Protein (SYPRO Ruby). ***B***, Quantification of MG-H1 immunoblot density normalized to SYPRO Ruby total protein stain. The sum of band density for the three bands indicated by arrowheads was used for analysis.

To test the hypothesis that accumulation of methylglyoxal disrupts AIS geometry, we first needed to determine the concentration of methylglyoxal required to disrupt cellular methylglyoxal metabolism in our *in vitro* cortical culture model. Reliable estimates of methylglyoxal concentration in the *in vivo* nervous system during normal, physiological conditions are 1.2 μm (Eberhardt et al., 2012) or 40 nmol/g (Liu et al., 2017) in control dorsal root ganglia, 0.3–1.5 μm (Kurz et al., 2011) or 5 μm (Distler et al., 2012) in young adult mouse brain, and 10 μm in the cerebrospinal fluid of healthy control middle-aged humans (Kuhla et al., 2005). Conversion of 40 nmol/g in Liu et al. (2017) to a micromolar concentration assuming a density of DRG tissue of ∼1 g/ml yields 40 μm methylglyoxal. Thus, these studies suggest the normal concentration of methylglyoxal in the brain could be 1–40 μm. Other studies report methylglyoxal levels in whole brain as high as 170–360 μm (Hambsch et al., 2010), but this may be an overestimation because of inappropriate sample processing (Rabbani and Thornalley, 2014). Information about methylglyoxal levels in CNS tissue during pathophysiological disease conditions such as type 2 diabetes are not well-established. Increased methylglyoxal concentration in the blood or serum of patients with type 2 diabetes (Andersen et al., 2018), Alzheimer’s disease (Haddad et al., 2019), and aging-related cognitive decline (Beeri et al., 2011) suggest that levels of methylglyoxal may increase in CNS tissues during neurological disease. To validate the concentration of methylglyoxal required to produce pathophysiological disruption of cellular methylglyoxal metabolism in our postnatal cortical cultures, we tested if 24 h exposure to 0, 1, 10, or 100 μm methylglyoxal would increase hydroimidazolone (MG-H1), a methylglyoxal-derived advanced glycation end-product (MG-AGE). MG-H1 is a suitable marker for disrupted cellular methylglyoxal metabolism since pathophysiological increase of methylglyoxal leads to accumulation of MG-AGEs *in vivo*, and upwards of 90% of MG-AGEs *in vivo* are the MG-H1 moiety (Rabbani and Thornalley, 2012). MG-H1 and Total Protein in immunoblots of postnatal cortical culture cell lysates are shown (Fig. 3*A*). MG-H1 relative band density (normalized to total protein lane volume) was increased by 100 μm methylglyoxal (*p *=* *0.001) but not 1 or 10 μm (*F*_(3,8)_ = 16.08; *p *=* *0.0009; Dunnett; *n* = 3 cultures; Fig. 3*B*). These results indicate that adding 100 μm methylglyoxal for 24 h is sufficient to acutely disrupt cellular methylglyoxal metabolism in cortical cultures, suggesting that this is a pathophysiologically-relevant dose in our *in vitro* model.

### Characterization of AIS geometry after methylglyoxal exposure

**Figure 4.**
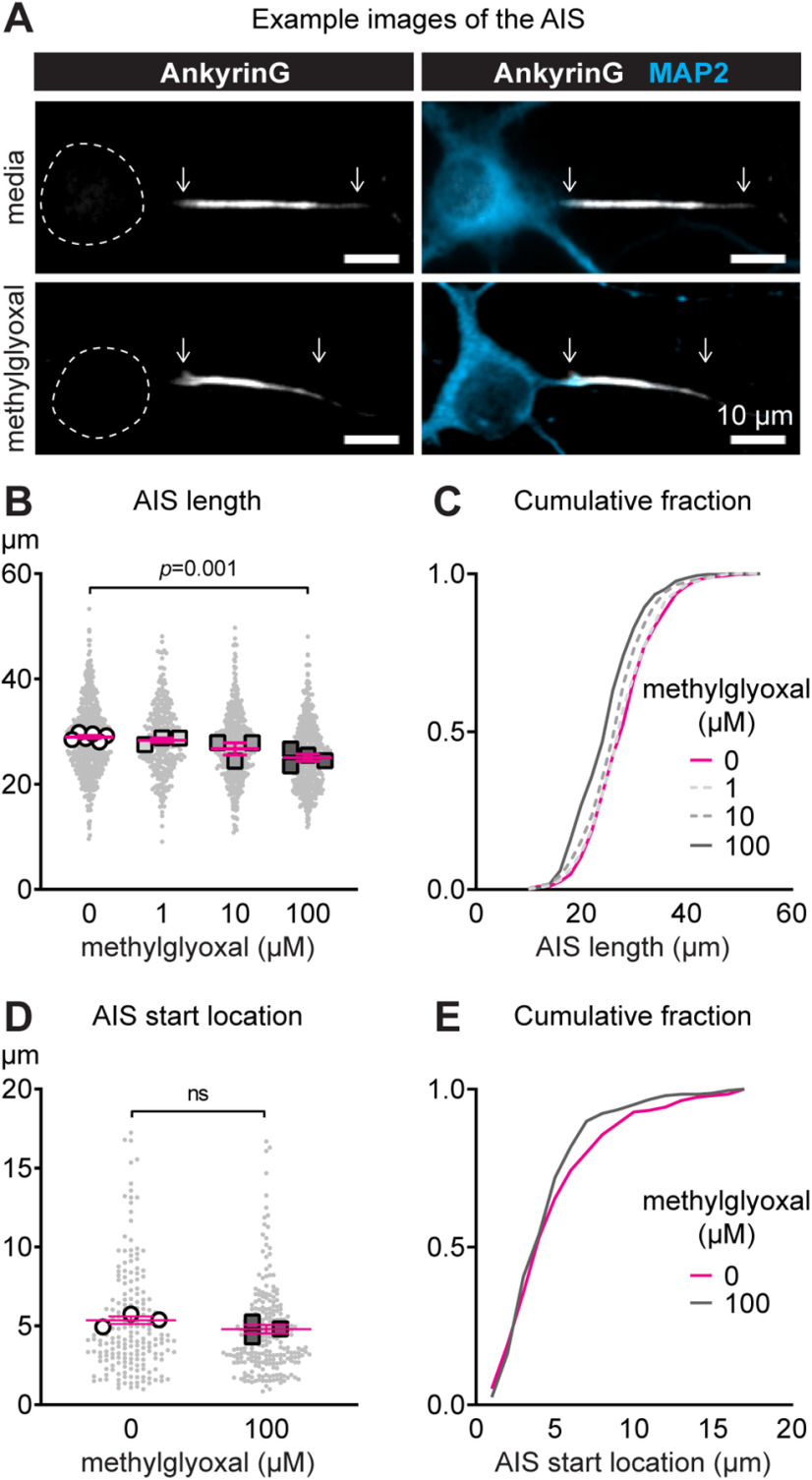
Methylglyoxal reduces AIS length in mouse cortical cultures. ***A***, AnkyrinG (AIS protein) and MAP2 (somatodendritic protein) immunofluorescence after 24 h of exposure to media or 100 μm methylglyoxal at DIV10–DIV11. Dashed lines indicate the neuronal soma. Scale bar: 10 μm. ***B***, Quantification of AIS length (AnkyrinG immunostaining) after 24 h of exposure to various concentrations of methylglyoxal (0–100 μm). Small gray dots indicate each AIS and larger open symbols represent the average AIS length for each culture preparation (326–598 AIS were analyzed from *n* = 3–6 cultures); *p* value indicates statistical comparison at the culture preparation level. ***C***, Cumulative fractional distribution of AIS length after 24 h of exposure to 0–100 μm methylglyoxal. ***D***, AIS start location (AnkyrinG immunostaining) after 24 h of exposure to 100 μm methylglyoxal. Small gray dots indicate each AIS and larger open symbols represent the average AIS start location for each culture preparation (194–246 AIS were analyzed from *n* = 3 cultures). ns (not significant) indicates statistical comparison at the culture preparation level. ***E***, Cumulative fractional distribution of AIS start location after 24 h of exposure to 100 μm methylglyoxal.

To test whether methylglyoxal shortens the AIS, we exposed mature neuronal networks to a range of concentrations (0–100 μm) *in vitro* that likely represent physiological to potentially pathophysiological levels for 24 h. Representative images of AnkyrinG and microtubule associated protein 2 (MAP2; somatodendritic marker) show a shorter AIS length after 100 μm methylglyoxal exposure for 24 h (Fig. 4*A*). AIS length after 24 h of exposure to 0 μm (media control; 28.79 ± 0.28 μm, *n* = 598 AIS from 6 cultures), 1 μm (28.39 ± 0.36 μm, *n* = 326 AIS from three cultures), 10 μm (27.26 ± 0.29 μm, *n* = 489 AIS from three cultures), or 100 μm (25.48 ± 0.26 μm, *n* = 522 AIS from four cultures) methylglyoxal indicate a dose-dependent inverse relationship between concentration of methylglyoxal and AIS length (slope = −1.47, *R*^2^ = 0.85, *p *<* *0.0001; linear regression analysis of means) with significant AIS shortening by 10 μm (*p *=* *0.0004, 5.3% reduction) and 100 μm (*p *< 0.0001, 11.5% reduction) methylglyoxal compared with media only (dose: *F*_(4,2159)_ = 63.60, *p *<* *0.0001; Dunnett). There was also significant AIS shortening by 100 μm (*p *=* *0.001) but not 10 μm (*p *=* *0.08) or 1 μm (*p* = 0.89) methylglyoxal when analyzed by culture preparation (dose: *F*_(3,12)_ = 9.66, *p *=* *0.002; Tukey; *n* = 3–6 cultures; Fig. 4*B*). This 11.5% AIS shortening induced by 100 μm methylglyoxal is about half of the 25% shortening induced by patterned optogenetic stimulation or high KCl (Evans et al., 2015) and similar to 8–16% decrease in AIS length in cortical neurons of db/db mice (Yermakov et al., 2018). AIS shortening by 100 μm methylglyoxal was generalized across the population of neurons, as indicated by a leftward shift in the cumulative frequency distribution (Fig. 4*C*).

In addition to AIS length changes, AIS relocation (change in the position of the AIS relative to the soma) is implicated in experimental demyelination (Hamada and Kole, 2015), epilepsy (Harty et al., 2013), and as a mechanism of activity-dependent plasticity that alters intrinsic neuronal function (Grubb and Burrone, 2010; Evans et al., 2013, 2017). Therefore, we tested whether exposure to 100 μm methylglyoxal for 24 h relocated the start location of the AIS. AIS start location after 24 h of exposure to media (5.23 ± 0.24 μm, *n* = 194 AIS from three cultures) or 100 μm methylglyoxal (4.70 ± 0.17, *n* = 246 AIS from three cultures) was similar when analyzed by AIS (*p *=* *0.07, unpaired homoscedastic two-tailed *t* test) or culture preparation (*p *=* *0.19, unpaired homoscedastic two-tailed *t* test; *n* = 3 cultures) as shown by scatter plot (Fig. 4*D*) or cumulative frequency distribution (Fig. 4*E*). This is consistent with no change in the AIS start location in db/db prefrontal cortex (Yermakov et al., 2018).

### Methylglyoxal-evoked AIS shortening is not associated with cell death and is reversible

**Figure 5.**
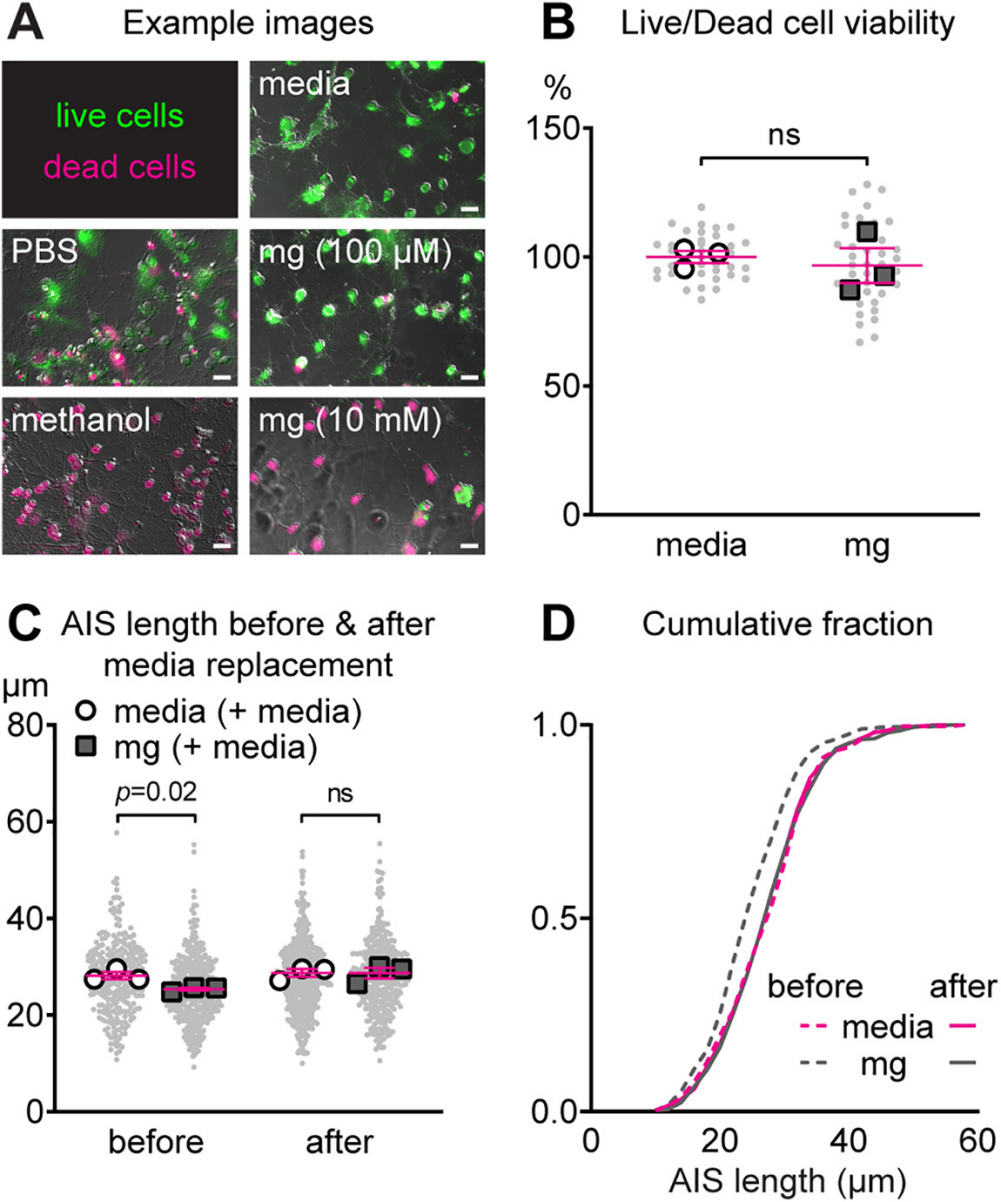
AIS shortening by methylglyoxal does not alter cell viability and is reversible. ***A***, Representative images of live/dead assay results after PBS (control) or methanol (to induce cell death) exposure for 15 min and after 24 h of exposure to media, 100 μm methylglyoxal, or 10 mm methylglyoxal. Scale bar: 20 μm. ***B***, Cell viability (live/dead assay shown as the % of live cells normalized to media control) results after 24 h of exposure to media or 100 μm methylglyoxal. Small gray dots indicate each image and larger open symbols represent the average % of live cells for each culture preparation (40 images were analyzed from *n* = 3 cultures). ns (not significant) indicates statistical comparison at the culture preparation level. ***C***, AIS length after an initial exposure to media or 100 μm methylglyoxal for 24 h (before), followed by replacement with conditioned media and a 24-h recovery period (after). Small gray dots indicate each AIS and larger open symbols represent the average AIS length for each culture preparation (274–499 AIS were analyzed from *n* = 3 cultures); *p* value or ns (not significant) indicates statistical comparison at the culture preparation level. ***D***, Cumulative fractional distribution of AIS length before and after media replacement following the initial 24 h of exposure to media or 100 μm methylglyoxal.

To rule out the possibility that AIS shortening by methylglyoxal is because of cellular injury we assessed cell viability after methylglyoxal exposure. Live/dead analysis of exposure to methanol, a positive control, or 24 h of exposure to a supraphysiological dose of 10 mm methylglyoxal showed substantial cell death (Fig. 5*A*). However, compared with media, 24 h of exposure to 100 μm methylglyoxal did not affect cell viability when analyzed at the image (*p *=* *0.33, unpaired homoscedastic *t* test; *n* = 40 images from three cultures) or culture (*p *=* *0.66, unpaired homoscedastic *t* test; *n* = 3 cultures) level (Fig. 5*B*). Our results are in agreement with the absence of cell death in cultured embryonic (E17) mouse cortical neurons at DIV14 (Bélanger et al., 2011), DRG neurons (Radu et al., 2012), or SH-SY5Y cells (Nishimoto et al., 2017) after *in vitro* exposure to 100 μm methylglyoxal for 24 h. Similarly *in vivo*, intraperitoneal injection of methylglyoxal increased the concentration of methylglyoxal in the brain without cytotoxicity in the hippocampus (Distler et al., 2012). In type 2 diabetic db/db mice at 10–11 weeks of age, there is elevated free methylglyoxal (Bierhaus et al., 2012) and MG-H1 (Griggs et al., 2019) in serum and AIS shortening without apoptotic cell death in the prefrontal cortex (Yermakov et al., 2018).

A previous study of AIS plasticity in embryonic rat hippocampal neurons indicates that AIS shortening initiated by high KCl is recoverable when cultures are returned to conditioned media for 24 h (Evans et al., 2015). Thus, we tested whether media replacement would lead to recovery of AIS shortening after an initial 24 h of exposure to 100 μm methylglyoxal. There was a significant interaction of AIS length between media or methylglyoxal exposure and before or after media replacement (drug by time interaction: *F*_(1,1496)_ = 10.48, *p *=* *0.001; Tukey). Before media replacement, methylglyoxal AIS length (25.46 ± 0.35 μm, *n* = 383 AIS from three cultures) was shorter compared with media (27.9 ± 0.46 μm, *n* = 274 AIS from 3 cultures; *p *=* *0.0002). Twenty-four hours after media replacement, AIS length was similar in cultures initially exposed to media (media + media, 27.92 ± 0.33 μm, *n* = 499 from three cultures) or methylglyoxal (methylglyoxal + media, 28.10 ± 0.40 μm, *n* = 343 from 3 cultures; *p *>* *0.99). Similarly at the culture preparation level, the AIS was shorter after the initial 24 h methylglyoxal exposure before (*p *=* *0.02; unpaired homoscedastic *t* test; *n* = 3 cultures) but not after (*p *=* *0.99; unpaired homoscedastic *t* test; *n* = 3 cultures) media replacement (Fig. 5*C*). Cumulative frequency distribution illustrates methylglyoxal-evoked AIS shortening before but not after media replacement (Fig. 5*D*).

### Methylglyoxal shortens the AIS in multiple cell types

**Figure 6.**
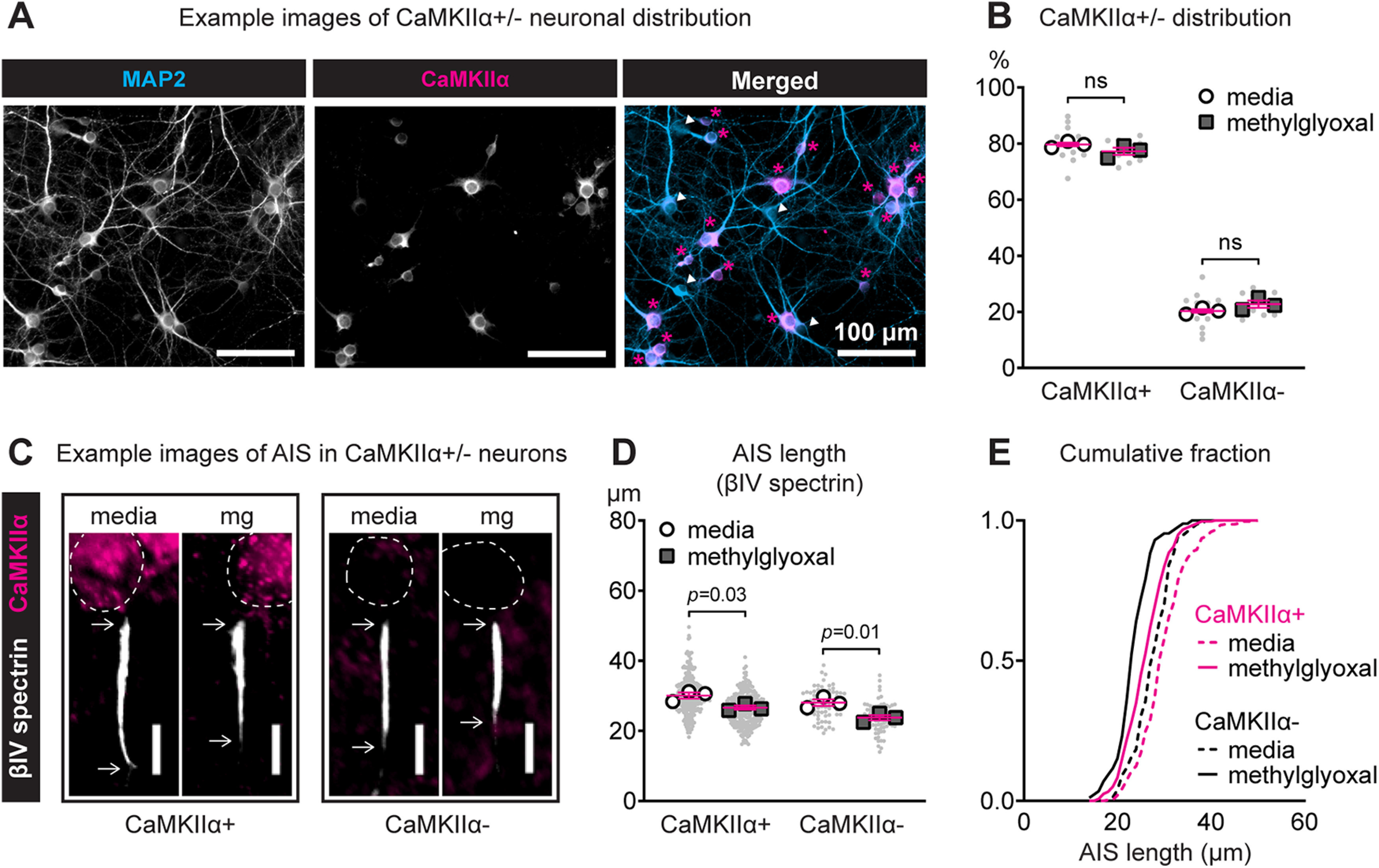
Methylglyoxal shortens the AIS in both excitatory and putative inhibitory neurons. ***A***, Representative images showing MAP2 (soma and dendrites) and CaMKIIα (excitatory neuron marker) immunostaining in DIV14 postnatal cortical cultures. CaMKIIα+ (magenta asterisks) and CaMKIIα– (white arrowheads) neurons are indicated in the merged image. Scale bar: 100 μm. ***B***, Quantification of the percentage of total cells counted that were CaMKIIα+ (excitatory) or CaMKIIα– (putative inhibitory) after 24 h of exposure to 100 μm methylglyoxal. Small gray dots indicate each coverslip and larger open symbols represent the average % of CaMKIIα+/− cells for each culture preparation (12 coverslips were analyzed from *n* = 3 cultures). ns (not significant) indicates statistical comparison at the culture preparation level. ***C***, Representative images of the AIS (βIV spectrin) in CaMKIIα+ and CaMKIIα– neurons after 24 h of exposure to 100 μm methylglyoxal. Arrows denote the start and end of the AIS. Dashed lines indicate the neuronal soma. Scale bar: 10 μm. ***D***, Quantification of AIS length (βIV spectrin) after 24 h of exposure to media or 100 μm methylglyoxal in CaMKIIα+ and CaMKIIα– neurons. Small gray dots indicate each AIS and larger open symbols represent the average AIS length for each culture preparation (278–298 AIS were analyzed from *n* = 3 cultures for CaMKIIα+, 76–86 AIS were analyzed from *n* = 3 cultures for CaMKIIα–); *p* values indicate statistical comparison at the culture preparation level. ***E***, Cumulative fractional distribution of AIS length in CaMKIIα+ and CaMKIIα– neurons after 24 h of exposure to 0–100 μm methylglyoxal.

Our results suggest that methylglyoxal produces AIS shortening in the entire population of cortical neurons (Figs. 4*C*, 5*D*), whereas previous studies indicate cell type-dependent AIS plasticity in hippocampal or olfactory bulb cultures (Grubb and Burrone, 2010; Evans et al., 2013, 2015; Chand et al., 2015). To test whether methylglyoxal produces AIS shortening in specific types of cortical neurons, we immunostained for MAP2 to label all neurons, βIV spectrin to label the AIS, and CaMKIIα to identify excitatory (CaMKIIα+) or putative inhibitory (CaMKIIα−) neurons. Representative images of postnatal cortical cultures at DIV14 double-labeled for MAP2 and CaMKIIα are shown (Fig. 6*A*). Exposure to 100 μm methylglyoxal for 24 h did not change the distribution of CaMKIIα+/− neurons: the mean percentage of all AIS measured that originated from CaMKIIα+ neurons was similar between media (79.7 ± 1.8%, 12 coverslips from three cultures) and methylglyoxal (77.3 ± 1.1%, 12 coverslips from three cultures) treatments when analyzed by coverslip (*p *=* *0.46; unpaired *t* test; *n* = 12 coverslips) or by culture (*p *=* *0.29; unpaired *t* test; *n* = 3 cultures; Fig. 6*B*). Analysis of only neurons treated with media control indicated that AIS length in CaMKIIα− (27.81 μm; *n* = 298) was ∼7.5% shorter than CaMKIIα+ (30.08 μm; *n* = 76) neurons. This shorter AIS length in inhibitory neurons is consistent with a previous study of hippocampal cultures from GAD67-GFP transgenic mice (Prestigio et al., 2019). Representative images of AIS length in CaMKIIα+ and CaMKIIα− neurons after 24 h of exposure to 100 μm methylglyoxal are shown (Fig. 6*C*). Methylglyoxal shortened the AIS in both CaMKIIα+ and CaMKIIα− neurons at the culture preparation level (drug: *F*_(1,8)_ = 24.27, *p *=* *0.001; *n* = 3 cultures). In CaMKIIα+ neurons, AIS length after methylglyoxal exposure (26.25 ± 0.27 μm, *n* = 278 AIS from three cultures) was shorter than media control (30.08 ± 0.30 μm, *n* = 298 AIS from three cultures) at both the AIS (*p *<* *0.0001; unpaired homoscedastic two-tailed *t* test, 12.7% reduction) and culture preparation level (*p *=* *0.03; Sidak; Fig. 6*D*). Similarly, in CaMKIIα− neurons, AIS length after methylglyoxal exposure (23.65 ± 0.42 μm, *n* = 86 AIS from three cultures) was shorter than media control (27.81 ± 0.47 μm, *n* = 76 AIS from three cultures) at both the AIS (*p *<* *0.0001, unpaired homoscedastic two-tailed *t* test, 15.0% reduction) and culture preparation level (*p *=* *0.01; Sidak; Fig. 6*D*). Cumulative frequency distribution of AIS length exemplifies the difference in AIS length between CaMKIIα+ and CaMKIIα− neurons after media control treatment as well as methylglyoxal-evoked AIS shortening in both CaMKIIα+ and CaMKIIα− neurons (Fig. 6*E*).

Taken together, our results using two complementary AIS markers, AnkyrinG (Figs. 4*A–C*, 5*C*,*D*) and βIV spectrin (Fig. 6*C–E*), indicate that increase of methylglyoxal, but not insulin resistance or high glucose alone (Figs. 1, 2), could be the key initiator of AIS shortening during type 2 diabetes. Exposure to 100, but not 1 or 10, μm methylglyoxal for 24 h was sufficient to increase cellular methylglyoxal to potentially pathophysiological levels (Fig. 3) and shorten the AIS (Fig. 4) in a reversible manner without cell death (Fig. 5) in both excitatory and inhibitory cortical neuron populations (Fig. 6). These results suggest that 100 μm methylglyoxal could be a pathophysiologically-relevant dose of methylglyoxal that leads to AIS shortening in our *in vitro* cortical culture model. We used this 100 μm dose to characterize the time course of methylglyoxal-evoked changes in cellular excitability, neuronal network activity, and AIS length to better understand the relationship between AIS geometry and neuronal network operation.

### Effect of methylglyoxal on cortical neuron resting membrane potential and excitability parameters

Subtle AIS shortening (a change in length as small as 4.5%) is reported to reduce neuronal excitability at the cellular (Baalman et al., 2013; Evans et al., 2015; Vascak et al., 2017; Goethals and Brette, 2020; Jamann et al., 2021) and network (Fruscione et al., 2018; Sohn et al., 2019) levels. In the next experiments we tested the hypothesis that methylglyoxal-evoked AIS shortening would be associated with reduced neuronal function at both the cellular and network levels.

**Figure 7.**
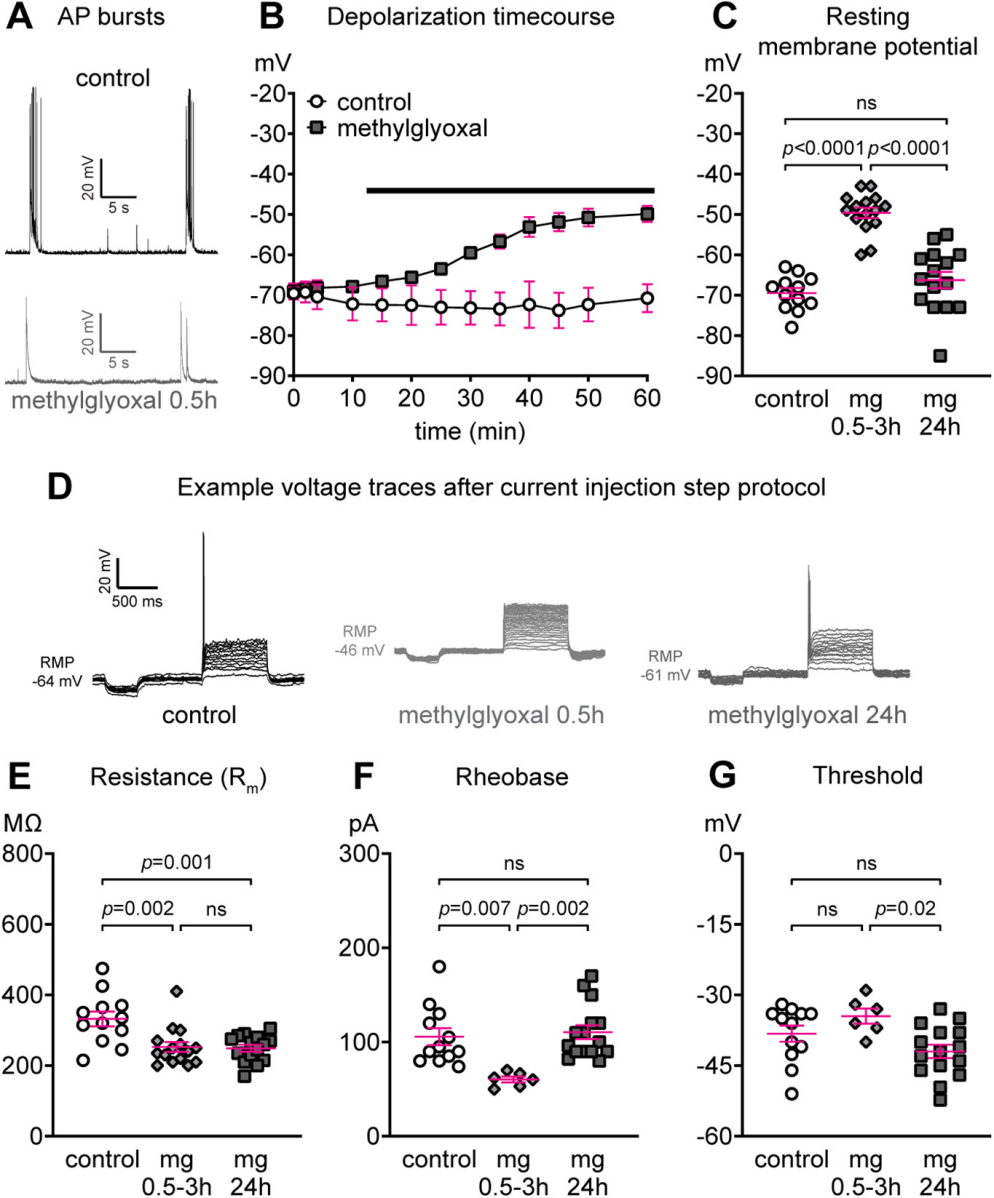
Depolarization of resting membrane potential by methylglyoxal. ***A***, Example traces of spontaneous action potentials (APs) in aCSF control (top) or 0.5 h after exposure to 100 μm methylglyoxal (bottom). ***B***, Time course of changes in resting membrane potential during exposure to aCSF control (*n* = 2 cells) or 100 μm methylglyoxal (*n* = 4 cells) conditions. Black bar indicates continuous infusion of aCSF only or aCSF containing 100 μm methylglyoxal. ***C***, Quantification of resting membrane potential in aCSF control (control; *n* = 12 cells from 6 coverslips) or at 0.5–3 h (*n* = 15 cells from 6 coverslips) or 24 h (*n* = 15 cells from 6 coverslips) after exposure to 100 μm methylglyoxal. Depolarization of resting membrane potential by methylglyoxal at 0.5–3 h recovered by 24 h. ***D***, Representative voltage traces during the current injection step protocol (500-ms duration, 10 pA per step) in aCSF control (14 steps) or at 0.5 h (27 steps) or 24 h (14 steps) after exposure to 100 μm methylglyoxal. The voltage traces are plotted on the same *y*-axis (data not shown), and the resting membrane potential for each cell is indicated. ***E***, Resistance of the membrane (R_m_) in aCSF control or at 0.5–3 h or 24 h after exposure to 100 μm methylglyoxal. ***F***, Rheobase (current threshold) in aCSF control or at 0.5–3 h or 24 h after exposure to 100 μm methylglyoxal. Note that only 6 out of 15 cells were able to fire APs at 0.5–3 h, whereas 15 out of 15 cells fired APs at 24 h. ***G***, Threshold potential in aCSF control or at 0.5–3 h or 24 h after exposure to 100 μm methylglyoxal. Note that only 6 out of 15 cells were able to fire APs at 0.5–3 h whereas 15 out of 15 cells fired APs at 24 h. ***C***, ***E***, ***F***, ***G***: *p* value or ns (not significant) indicates statistical comparison at the cellular level.

Previous studies indicate that depolarization by high KCl leads to rapid AIS shortening within 1–3 h (Evans et al., 2015; Jamann et al., 2021) and methylglyoxal (100–300 μm) depolarizes the resting membrane potential in rat somatosensory cortex slices within minutes (de Arriba et al., 2006) and in mouse DRG neurons within 3 h (Bierhaus et al., 2012). Taken together, these studies suggest that depolarization may play a role in AIS shortening by methylglyoxal. To determine the time course of changes in resting membrane potential in our cortical cultures, we performed whole-cell patch clamp recordings from pyramidal-like neurons after exposure to 100 μm methylglyoxal. We first recorded resting membrane potential changes in the same cell before and after continuous infusion of aCSF alone or aCSF containing 100 μm methylglyoxal. Example traces of spontaneous spiking suggest that methylglyoxal may reduce action potential bursts within 0.5 h compared with control (Fig. 7*A*). Methylglyoxal caused a gradual depolarization from −69.1 ± 2.0 mV (*t* = 0 min) to −49.9 ± 2.0 (*t* = 60 min; *n* = 4 cells from four coverslips) whereas control aCSF did not change the resting membrane potential (*n* = 2 cells from 2 coverslips; Fig. 7*B*). This ∼19 mV depolarization by methylglyoxal is similar to that reported in previous studies (de Arriba et al., 2006; Bierhaus et al., 2012). In the second set of experiments, we assessed resting membrane potential input resistance, rheobase, and voltage threshold in cultured cortical neurons during control conditions (aCSF only) or after exposure to 100 μm methylglyoxal for 0.5–3 or 24 h using a current injection step protocol. Resting membrane potential was depolarized by methylglyoxal (drug; *F*_(2,39)_ = 44.18, *p *<* *0.0001; Tukey) at 0.5–3 h (49.6 ± 1.3 mV; *n* = 15 neurons from 6 coverslips) compared with control (−69.5 ± 1.3 mV; *n* = 12 neurons from 6 coverslips; *p *<* *0.0001 vs 0.5–3 h) but returned to normal at 24 h (−66.3 ± 2.0 mV; *n* = 15 neurons from 6 coverslips; *p *=* *0.36 vs control; Fig. 7*C*). Example traces of the step current protocol are consistent with depolarization-induced action potential blockade (Fig. 7*D*). Methylglyoxal reduced the input resistance (drug; *F*_(2,39)_ = 9.19, *p *=* *0.0005; Tukey) at 0.5–3 h (252.7 ± 14 MΩ; *p *=* *0.002 vs control) and at 24 h (249.0 ± 9.9 MΩ; *p *=* *0.001 vs control) compared with the control (332 ± 21 MΩ; Fig. 7*E*). Threshold current necessary to trigger an action potential (rheobase) was reduced by methylglyoxal (drug; *F*_(2,30)_ = 7.66, *p *=* *0.002; Tukey) at 0.5–3 h (60.2 ± 3.1 pA; *n* = 6 cells from 6 coverslips; *p *=* *0.007 vs control) compared with the control (105.8 ± 9.1 pA; *n* = 12 cells from 6 coverslips) and returned to normal at 24 h (110.5 ± 7.4 pA; *n* = 15 cells from 6 coverslips; *p *=* *0.89 vs control; Fig. 7*F*). Methylglyoxal slightly altered the threshold potential (drug; *F*_(2,30)_ = 4.41, *p *=* *0.02; Tukey) with a small but non-significant increase at 0.5–3 h (−34.5 ± 1.6 mV; *n* = 6 cells from 6 coverslips; *p *=* *0.37 vs control) and a slight reduction at 24 h (−42.0 ± 1.4 mV; *n* = 15 cells from 6 coverslips; *p *=* *0.02 vs 0.5–3 h) compared with the control (−38.2 ± 1.7 mV; *n* = 12 cells from 6 coverslips; *p *=* *0.19 vs 24 h; Fig. 7*G*). Importantly, 7 out of 15 cells at 0.5–3 h were unable to fire even with up to 260 pA current injection, possibly because depolarization of resting membrane potential caused inactivation of sodium channels.

### Effect of methylglyoxal on neuronal network activity

**Figure 8.**
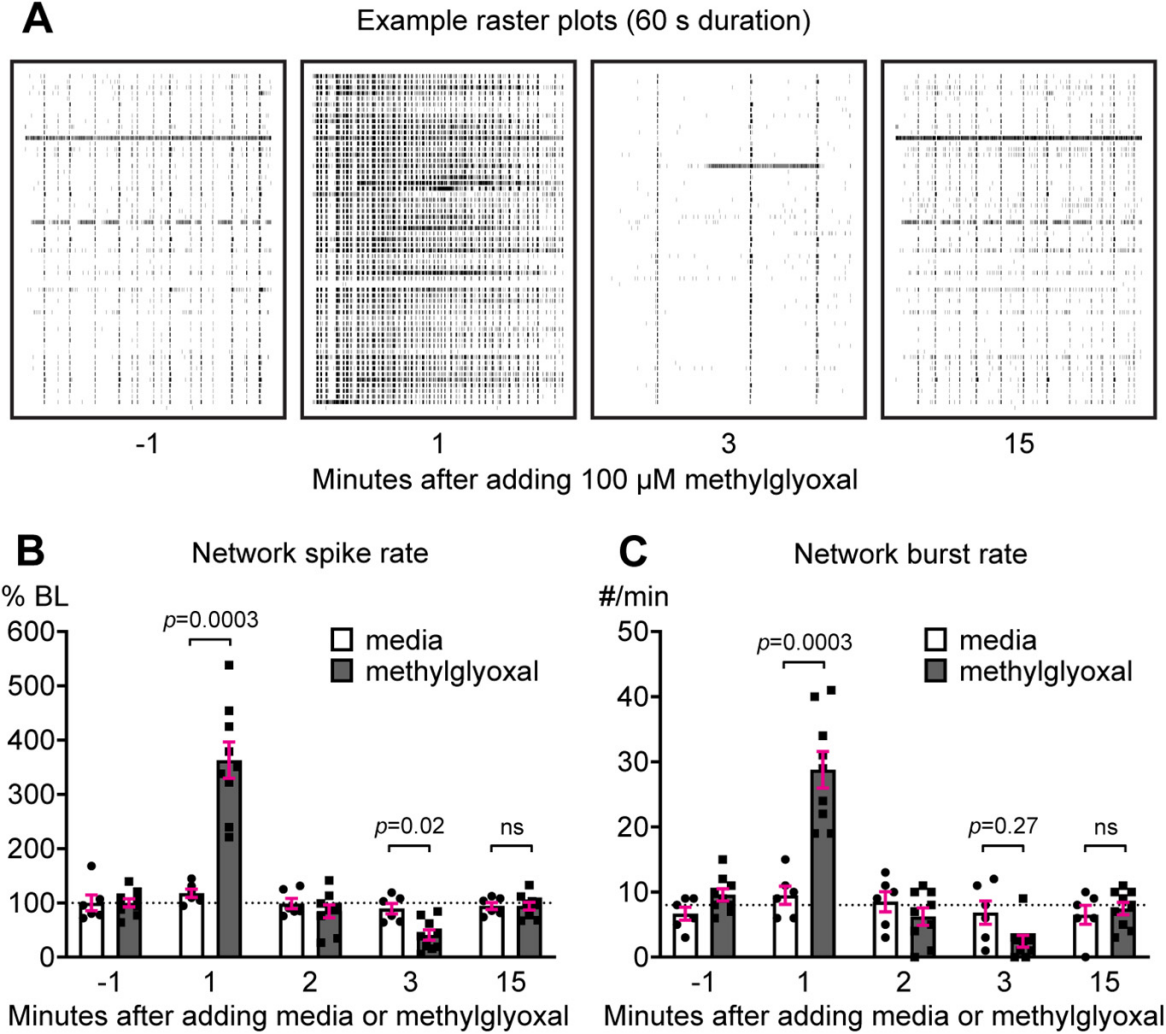
Methylglyoxal transiently increases neuronal network activity within the first few minutes. ***A***, Representative raster plots (60-s duration) of MEA recordings showing spike activity (60 electrodes, one per row) during 1-min intervals before and after exposure to 100 μm methylglyoxal. ***B***, Quantification of network spike rate (normalized as percent of −1-min baseline) during 1-min intervals before and after exposure to media or 100 μm methylglyoxal (*n* = 6 MEAs from 2 cultures for media and *n* = 9 MEAs from 3 cultures for methylglyoxal). Dashed line indicates baseline. ***C***, Quantification of network burst rate (MEA-wide bursts per minute) during 1-min intervals before and after exposure to media or 100 μm methylglyoxal (*n* = 6 MEAs from 2 cultures for media and *n* = 9 MEAs from 3 cultures for methylglyoxal). Dashed line indicates baseline.

Our results (Fig. 7) and previous studies indicate that methylglyoxal acutely alters neuronal activity at the cellular level (de Arriba et al., 2006; Bierhaus et al., 2012; Radu et al., 2012; Andersson et al., 2013; Griggs et al., 2019). To determine the effect of methylglyoxal on the coordinated activity of neuronal networks we used MEA recordings of our cortical cultures. Characterization of MEA network activity before drug exposure revealed that MEA-wide network spike frequency increased from DIV7 (44.5 ± 4.8 Hz) to DIV13 (230.6 ± 10.9 Hz; *p *<* *0.0001; *n* = 9 MEAs from three cultures). Example sixty second raster plots from DIV13 show that adding 100 μm methylglyoxal resulted in an immediate increase of network activity, followed by a short period of quiescence, and restoration to baseline activity within 15 min (Fig. 8*A*). Methylglyoxal altered network spike rate compared with media control (drug × time interaction: *F*_(4,52)_ = 31.16; *p *<* *0.0001; *n* = 6 MEAs from 2 cultures for media control and *n* = 9 MEAs from three cultures for methylglyoxal; Sidak; Fig. 8*B*). Methylglyoxal increased network spike rate (363.0 ± 33.5%) compared with media control (117.7 ± 7.5%) during the first minute by ∼300%. At 3 min, methylglyoxal reduced network spike rate (40.9 ± 9.5%) to ∼50% of media control (89.3 ± 9.6%). At 15 min, network spike rates were similar to baseline after either methylglyoxal (*p *=* *0.99) or media (*p *=* *0.73) exposure. Similarly, methylglyoxal increased the network burst rate (number of MEA-wide network bursts per minute) immediately after its addition with restoration of baseline bursting at 15 min (drug × time interaction: *F*_(4,52)_ = 24.45; *p *<* *0.0001; Sidak; Fig. 8*C*).

**Figure 9.**
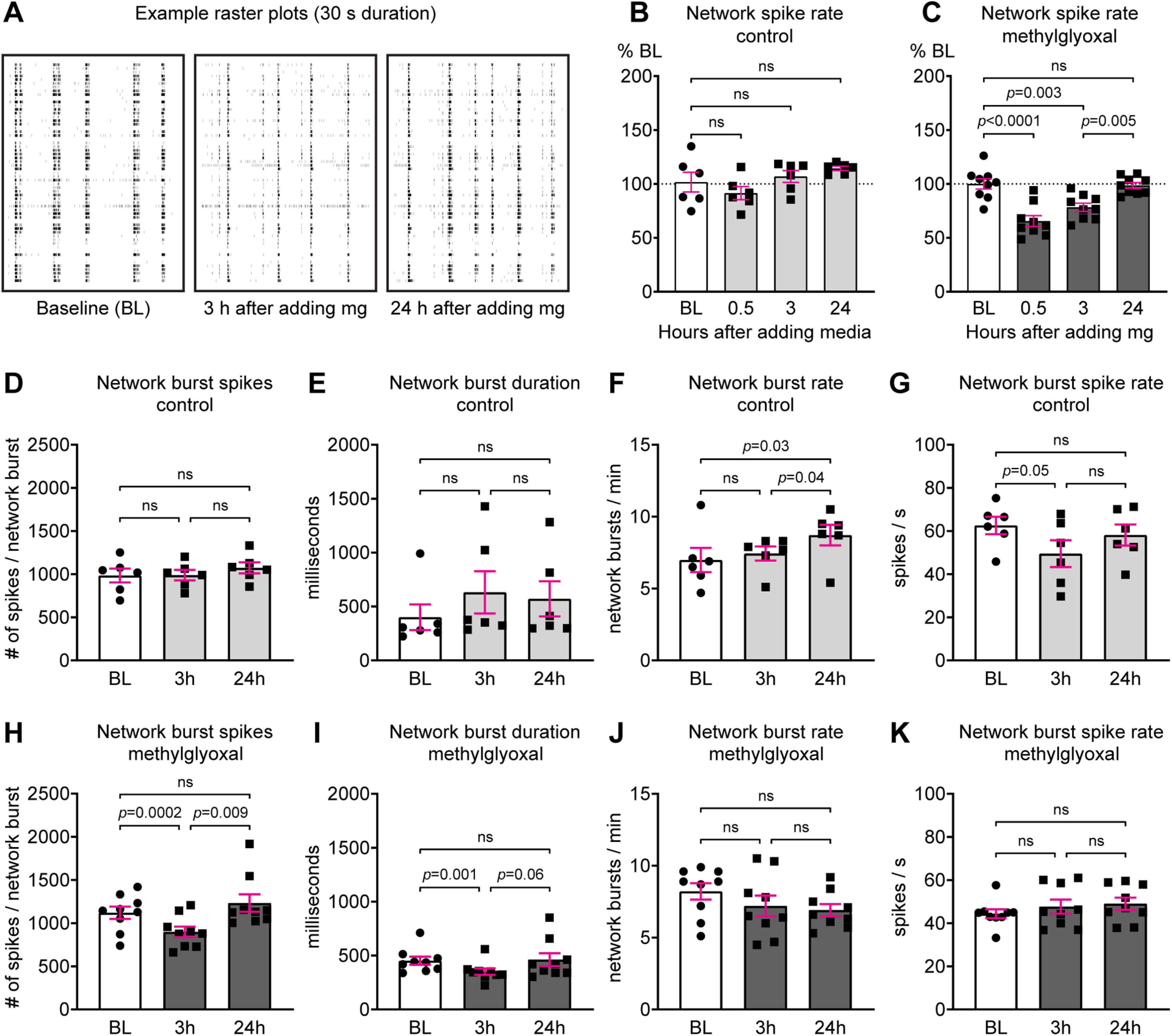
Reduction of neuronal network activity by methylglyoxal at 0.5–3 h recovers by 24 h. ***A***, Representative raster plots (30-s duration) showing spike activity (60 electrodes, one per row) during 15-min MEA recordings at baseline (BL) and 3 and 24 h after exposure to 100 μm methylglyoxal. ***B***, Network spike rate after media control exposure (*n* = 6 MEAs from 2 cultures). ***C***, Network spike rate after methylglyoxal exposure (*n* = 9 MEAs from 3 cultures). ***D–G***, Network spiking parameters after media control exposure. ***H–K***, Network spiking parameters after methylglyoxal exposure. ***B–K***: *p* values or ns (not significant) indicate statistical comparison at the MEA level.

We also recorded from the same MEAs at later timepoints to examine how cellular depolarization at 0.5–3 h (Fig. 7*C*) or AIS shortening at 24 h (Figs. 4-6) affect neuronal network activity. Example raster plots of neuronal network activity are shown at baseline and at 3 and 24 h after exposure to 100 μm methylglyoxal (Fig. 9*A*). Control exposure to media alone did not alter network spike rate (repeated measures across time: *F*_(3,15)_ = 1.92, *p *=* *0.17; *n* = 6 MEAs from 2 cultures; Fig. 9*B*). Methylglyoxal altered network spike rate (repeated measures across time: *F*_(3,24)_ = 18.9, *p *<* *0.0001; *n* = 9 MEAs from three cultures), with reduced spiking at 0.5 and 3 h that returned to baseline level at 24 h (Fig. 9*C*). Media control did not alter network burst spikes (number of spikes within network bursts; Fig. 9*D*) or network burst duration (Fig. 9*E*). Network burst rate (number of network bursts per min) in media control MEAs slightly increased during the experiment (Fig. 9*F*). Network burst spike rate (number of spikes per second within a network burst) was lower at 3 h and unchanged at 24 h by media control treatment (Fig. 9*G*). Methylglyoxal altered network burst spikes (Fig. 9*H*) and network burst duration (Fig. 9*I*) at 3 h without changing the network burst rate (Fig. 9*J*) or network burst spike rate (Fig. 9*K*).

### Spiking activity is not required for methylglyoxal-evoked AIS shortening

**Figure 10.**
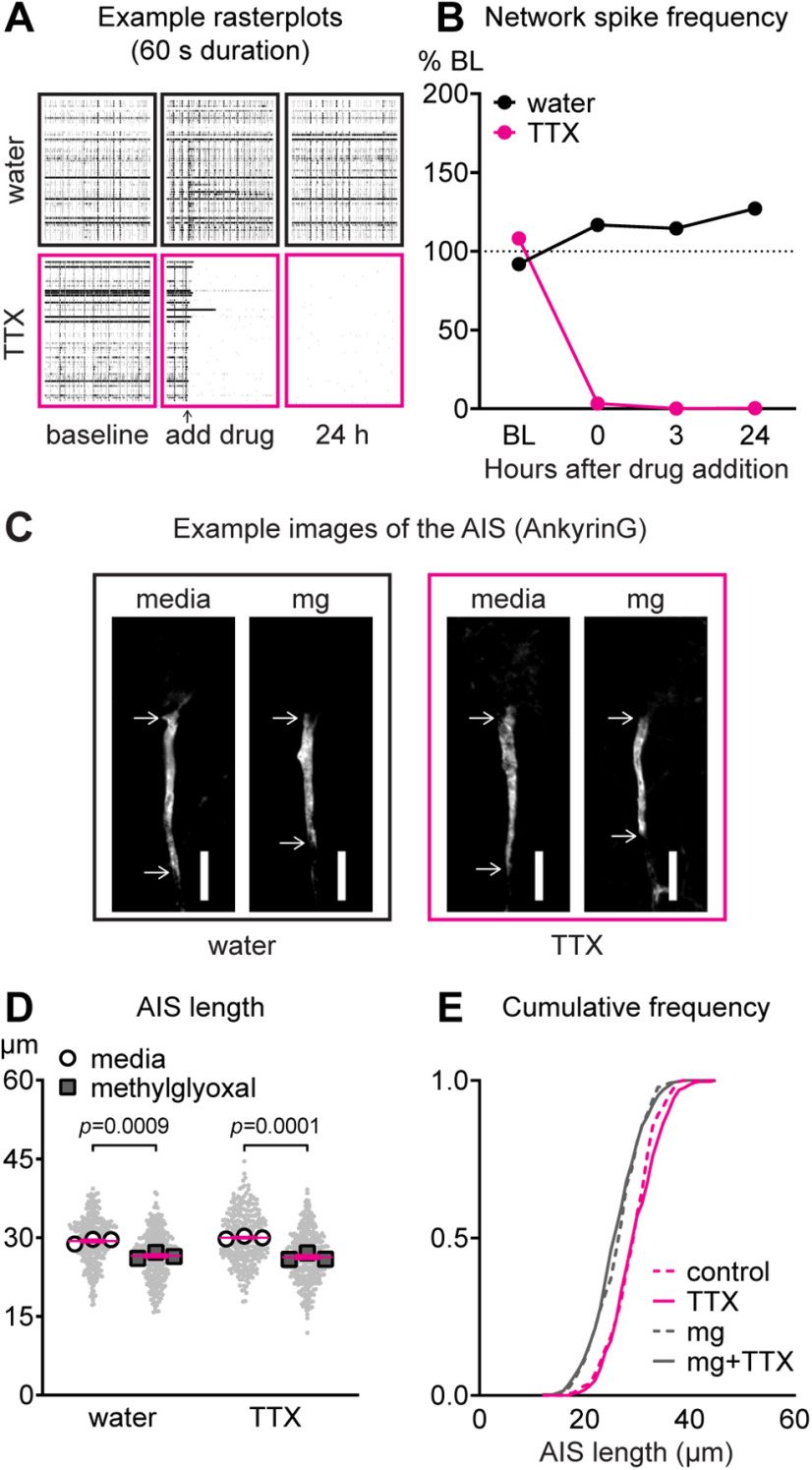
Methylglyoxal-evoked AIS shortening is independent of spiking activity. ***A***, Representative raster plots (60-s duration) showing spike activity (60 electrodes, one per row) at baseline, after adding drug (water or 1 μm TTX), and at 24 h. ***B***, Quantification of network spike rate (normalized as percent of baseline) before (BL) and after exposure to water or 1 μm TTX (*n* = 1 MEA). Dashed line indicates baseline average. ***C***, Representative images of AnkyrinG (AIS) immunostaining 24 h after exposure to media or 100 μm methylglyoxal alone or in combination with water or 1 μm TTX. Scale bar: 10 μm. ***D***, AIS length 24 h after co-exposure to media or methylglyoxal plus water or TTX indicating that TTX does not alter methylglyoxal-evoked AIS shortening. Small gray dots indicate each AIS and larger open symbols represent the average AIS length for each culture preparation (295–335 AIS were analyzed from *n* = 3 cultures); *p* values indicate statistical comparison at the culture preparation level. ***E***, Cumulative fractional distribution of AIS length after 24 h of exposure to methylglyoxal+TTX.

Our results indicate that reduction of network activity by methylglyoxal at 0.5–3 h returned to normal at 24 h (Fig. 9*C*) when we observed a shorter AIS (Figs. 4-6). This might suggest that AIS shortening is a homeostatic response to transient changes (increase at 1 min followed by decrease at 0.5–3 h) in network spiking and bursting. However, previous studies of AIS plasticity indicate that action potential spiking is not required for high KCl-evoked relocation (Grubb and Burrone, 2010) or shortening (Evans et al., 2015). To test whether methylglyoxal-evoked AIS shortening depends on the biphasic changes in spiking activity uncovered by MEA recordings (Figs. 8, 9), we co-exposed our cortical cultures to 100 μm methylglyoxal plus the sodium channel blocker TTX (1 μm) for 24 h and assessed spiking activity via MEAs and AIS length via AnkyrinG immunostaining. Example 60 s duration rasterplots (Fig. 10*A*) and quantification of network spike frequency (*n* = 1 MEA; Fig. 10*B*) show spiking and network bursting at baseline are almost completely abolished within seconds of adding 1 μm TTX whereas network activity after addition of water control is relatively unchanged. Elimination of network activity by TTX persisted for at least 24 h, when AIS length was quantified. Representative images after co-exposure to methylglyoxal and TTX are shown (Fig. 10*C*). Compared with media control, methylglyoxal shortened the AIS when co-exposed with water or TTX at both the AIS (*p *<* *0.0001; unpaired homoscedastic *t* test; *n* = 295–335 AIS from three cultures) and culture preparation level (main effect of methylglyoxal; *F*_(1,8)_ = 110.4, *p *<* *0.0001; *n* = 3 cultures; Fig. 10*D*). Importantly, AIS length was similar after co-exposure to media+water (control) or media+TTX (*p *=* *0.49; Tukey; *n* = 3 cultures) and after co-exposure to methylglyoxal+water or methylglyoxal+TTX (*p *=* *0.93; Tukey; *n* = 3 cultures). Cumulative frequency distribution of AIS length exemplifies the methylglyoxal-evoked AIS shortening in the presence or absence of TTX (Fig. 10*E*). These results indicate that neuronal spiking (action potentials) is not required for methylglyoxal-evoked AIS shortening, even though methylglyoxal temporarily changes network activity (Figs. 8,9). In addition, they show that almost complete elimination of spontaneous network activity by TTX alone does not change AIS length at 24 h.

### Time course of AIS shortening by methylglyoxal

**Figure 11.**
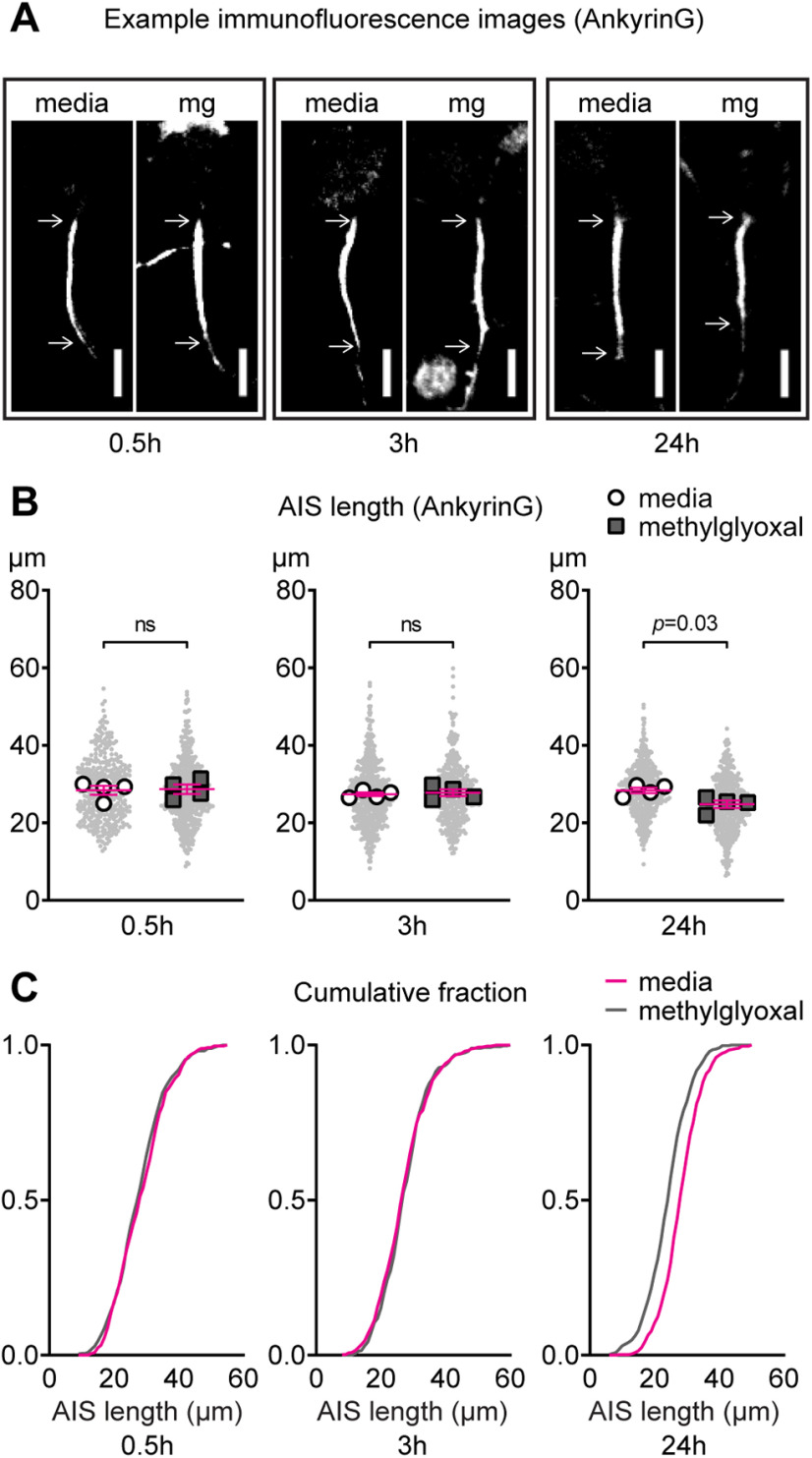
Time course of AIS shortening by methylglyoxal. ***A***, Representative images of AnkyrinG (AIS) immunostaining at 0.5, 3, 24 h after media or 100 μm methylglyoxal exposure. Scale bar: 10 μm. ***B***, AIS length at 0.5, 3, 24 h after media control treatment indicating AIS shortening at 24 h but not 0.5 or 3 h. Small gray dots indicate each AIS and larger open symbols represent the average AIS length for each culture preparation (377–594 AIS were analyzed from *n* = 4 cultures); *p* values or ns (not significant) indicate statistical comparison at the culture preparation level. ***C***, Cumulative fractional distribution of AIS length after 0.5, 3, and 24 h of exposure to methylglyoxal.

Our findings at 24 h after methylglyoxal exposure (Figs. 4, 7, 9) refute our initial hypothesis that AIS shortening would coincide with neuronal depolarization and reduced network activity. However, since we observed depolarization and reduced network activity at 0.5–3 h after methylglyoxal exposure (Figs. 7,9), and depolarization by high KCl leads to AIS shortening as rapid as 1 h in somatosensory cortex slices (Jamann et al., 2021) and 3 h in hippocampal neuron cultures (Evans et al., 2015), we checked AIS length at earlier timepoints. Representative images of AIS length after 0.5, 3, and 24 h of exposure to media or methylglyoxal are shown (Fig. 11*A*). AIS length at 0.5 h was similar when analyzed at both the AIS (*p *=* *0.20; unpaired homoscedastic *t* test; *n* = 377–530 AIS from four cultures) and culture preparation (*p *=* *0.86; unpaired homoscedastic *t* test; *n* = 4) levels (Fig. 11*B*). AIS length at 3 h was also similar at both the AIS (*p *=* *0.38; unpaired homoscedastic *t* test; *n* = 397–556 from four cultures) and culture preparation (*p *=* *0.70; unpaired homoscedastic *t* test; *n* = 4) levels (Fig. 11*B*). At 24 h, AIS length was reduced at both the AIS (*p *<* *0.0001; unpaired homoscedastic *t* test; *n* = 594–531 AIS from four cultures) and culture preparation (*p *=* *0.03; unpaired homoscedastic *t* test; *n* = 4) levels (Fig. 11*B*), confirming our previous results (Figs. 4-6). Cumulative frequency distributions illustrate methylglyoxal-evoked AIS shortening at 24 h but not at 0.5 or 3 h (Fig. 11*C*).

Contrary to AIS shortening within 1–3 h by high KCl (Evans et al., 2015; Jamann et al., 2021), we did not observe AIS shortening within 0.5–3 h of exposure to 100 μm methylglyoxal (Fig. 11), although methylglyoxal depolarized the neurons within this short 0.5 to 3 h time frame (Fig. 7*B*,*C*). Instead, we observed that AIS shortening by methylglyoxal was associated with normalization of resting membrane potential (Fig. 7*C*) and MEA network spiking (Fig. 9*C*) at 24 h. These observations, along with AIS shortening in both CaMKIIα+ and CaMKIIα− neurons (Fig. 6), hint that AIS shortening by methylglyoxal is likely different from homeostatic, cell type-dependent AIS shortening after depolarization by KCl.

## Discussion

### Methylglyoxal mediates AIS shortening

Because of the association between AIS shortening and cognitive impairment in type 2 diabetes we sought to identify the initiator of AIS shortening. Canonical features of type 2 diabetes, insulin resistance (Fig. 1) or high glucose (Fig. 2), did not dramatically alter AIS length in mouse cortical neuron cultures. In contrast, increase of methylglyoxal (100 μm) produced 11.5% AIS shortening (Fig. 4) that was recoverable and not associated with cell death (Fig. 5). This is consistent with 8–16% AIS shortening in the prefrontal cortex of type 2 diabetic db/db mice at 10 weeks of age with lack of apoptotic cell death (Yermakov et al., 2018). Methylglyoxal elevation leads to accumulation of MG-H1 in patients with type 2 diabetes (Schalkwijk and Stehouwer, 2020) and both methylglyoxal (Bierhaus et al., 2012) and MG-H1 (Griggs et al., 2019) are elevated in db/db mice that also display AIS shortening and cognitive impairment (Yermakov et al., 2018, 2019). Adding 100 μm methylglyoxal to culture media increased MG-H1 *in vitro* (Fig. 3), indicating that our culture model recapitulates pathophysiological disruption of methylglyoxal metabolism that might be relevant to diabetes. These results suggest that methylglyoxal is a key mediator of AIS shortening in the type 2 diabetic brain. However, we recognize the reductionist *in vitro* approach herein cannot completely recapitulate chronic diabetic conditions *in vivo*. We based our study on the limited information available in the literature suggesting 1–40 μm is the normal, physiological range of methylglyoxal in CNS tissues (Kuhla et al., 2005; Kurz et al., 2011; Distler et al., 2012; Eberhardt et al., 2012; Rabbani and Thornalley, 2014; Liu et al., 2017). Since cellular levels of methylglyoxal in the brain during neurological disease are not established, it is possible that the 100 μm methylglyoxal required to increase cellular MG-H1 and produce AIS shortening in the current *in vitro* study does not represent pathophysiological levels *in vivo.* Nevertheless, it is reasonable to think that prolonged increase of methylglyoxal, or the combined disruption of methylglyoxal, insulin, and glucose metabolism, during type 2 diabetes could result in sustained AIS shortening and CNS dysfunction.

### Methylglyoxal alters neuronal function at the cellular and network levels

Previous studies indicate that concentrations of methylglyoxal >1 mm were required to generate rapid inward currents in TRPA1-expressing HEK293 cells (Eberhardt et al., 2012) and mobilize calcium in DRG (Eberhardt et al., 2012) or spinal cord dorsal horn (Griggs et al., 2019) neurons. We observed that a much lower dose of 100 μm methylglyoxal rapidly changed both cellular and network function in our cortical cultures. At the network level, MEA spiking and bursting were increased for ∼1 min followed by a quick recovery to baseline within 15 min (Fig. 8). At the single-cell level, depolarization of resting membrane potential occurred within minutes, persisted for at least 3 h, and was associated with decreased rheobase and input resistance (Fig. 7). This is consistent with depolarization by 300 μm methylglyoxal in neocortical somatosensory neurons within minutes (de Arriba et al., 2006) or depolarization and decrease of rheobase in DRG neurons by 100 μm methylglyoxal exposure for 3 h (Bierhaus et al., 2012). The depolarization in our study (Fig. 7) coincided with a reduction of network spike rate and number of spikes per network burst (Fig. 9), suggesting that decreased network activity by methylglyoxal at 3 h might be because of depolarizing block of sodium channels. Interestingly, except for input resistance, these changes in cellular and network function recovered by 24 h despite the presence of a shorter AIS.

### How does methylglyoxal trigger AIS shortening and alter neuronal function?

The detailed mechanisms underlying methylglyoxal-evoked changes in network operation and AIS geometry in the current study remain unknown. Depolarization might contribute to the reduction of network activity at 0.5–3 h in addition to its potential role in AIS shortening (discussed below). Other explanations for altered neuronal function by methylglyoxal include decreased glutamate uptake (de Arriba et al., 2006; Lissner et al., 2021), depletion of mitochondrial ATP production (de Arriba et al., 2006, 2007), changes in the biophysical properties of sodium channels (Bierhaus et al., 2012), the cation channel transient receptor potential ankyrin A (TRPA1; Eberhardt et al., 2012; Andersson et al., 2013), or GABA_A_ receptors (Distler et al., 2012). Potential intracellular effectors of methylglyoxal-evoked AIS shortening include calcium, calpain, calcineurin, or tau. Methylglyoxal promotes calcium mobilization in neurons (Radu et al., 2012; Griggs et al., 2019), activation of calpain (Griggs et al., 2018) and calcineurin (Maeta et al., 2005), and hyperphosphorylation of tau (Li et al., 2012). Likewise, calcium channels (Grubb and Burrone, 2010; Evans et al., 2013, 2015; Chand et al., 2015), calpain (Schafer et al., 2009; Clark et al., 2017), calcineurin (Evans et al., 2015), and tau (Sohn et al., 2019) are all implicated in AIS disruption. Another intriguing candidate is hyperpolarization-activated cyclic nucleotide–gated (HCN) channels. Methylglyoxal increases HCN channel current (Shimatani et al., 2015) and activation of HCN channels specifically within the AIS is reported to decrease spike probability (Ko et al., 2016). This could explain the decreased spiking activity at 0.5–3 h (Fig. 9). Future studies are needed to determine whether the mechanisms described above are involved in the changes in neuronal function and AIS geometry that occur when methylglyoxal metabolism is disrupted.

### Are AIS geometry and neuronal function linked?

Several previous studies informed our initial hypothesis that AIS shortening would lead to reduced single-cell excitability and neuronal network activity. At the single-cell level, AIS shortening dampens multiple spike firing and increases firing threshold *in vitro* (Evans et al., 2015) and *in vivo* (Jamann et al., 2021). *In silico,* AIS shortening is associated with increase of interspike interval (Baalman et al., 2013), voltage threshold (Baalman et al., 2013; Goethals and Brette, 2020), and action potential acceleration (Vascak et al., 2017) in single neurons. These findings at the cellular level are consistent with shorter AIS length and reduced MEA burst rate at the network level (Fruscione et al., 2018; Sohn et al., 2019). By contrast, we observed depolarization of resting membrane potential, decreased rheobase, increased voltage threshold (Fig. 7) and reduced network spike and burst rates (Fig. 9) were not associated with shorter AIS length at 3 h (Fig. 11). Instead, the AIS was shorter at 24 h when single-cell excitability parameters and network activity recovered to baseline levels. These surprising findings might be explained by studies showing that AIS shortening does not reduce excitability when associated with changes in Nav phosphorylation (Evans et al., 2015), loss of Kv1 channels (Sanders et al., 2020), or alteration of action potential backpropagation (Radecki et al., 2018). Determining how simultaneous AIS shortening in multiple cell types and recovery of neuronal function are related will be critical going forward.

Differences in the pattern of AIS shortening and altered neuronal function elicited by methylglyoxal versus KCl highlight the importance of determining how the AIS and neuronal network operation are altered by pathophysiologically-relevant factors. AIS shortening has been described as a homeostatic mechanism that reduces neuronal excitability in response to depolarization by elevating KCl (Evans et al., 2015). In contrast, we did not observe concurrent AIS shortening (Fig. 11) and reduced network activity (Fig. 9). It is tempting to conclude that AIS shortening at 24 h is a homeostatic response to the transient changes in network activity (Figs. 8,9). However, methylglyoxal-evoked AIS shortening occurred even when action potentials were blocked by TTX (Fig. 10). Moreover, addition of TTX alone blocked network activity without changing AIS length (Fig. 10). These results are consistent with the idea that AIS plasticity is independent from network spiking activity (Grubb and Burrone, 2010; Evans et al., 2015). Depolarization is suggested to be a key factor in the rapid AIS shortening induced by high KCl at 3 h (Evans et al., 2015). Depolarization may contribute to AIS shortening in the current results, since it preceded AIS shortening, but the temporal relationship of depolarization and AIS shortening by methylglyoxal is different compared with increase of KCl. Although resting membrane potential was depolarized at 0.5–3 h (Fig. 7), we did not observe rapid AIS shortening by methylglyoxal at 0.5–3 h (Fig. 11). Instead, we observed AIS shortening at 24 h (Fig. 11) when resting membrane potential recovered to normal (Fig. 7). Another difference in the pattern of AIS shortening by methylglyoxal compared with KCl is the cell types involved. Evans et al. (2015) described cell-type specific AIS shortening in excitatory CA3 but not inhibitory GABAergic hippocampal neurons after 3 h of exposure to KCl. We observed cell-type independent AIS shortening in both excitatory CaMKIIα+ and putative inhibitory CaMKIIα− cortical neurons after 24 h of exposure to methylglyoxal (Fig. 6). Our observations hint that the mechanistic underpinnings of AIS shortening by methylglyoxal in cortical neurons are different from homeostatic AIS shortening by KCl in hippocampal neurons.

If AIS shortening by methylglyoxal is not a mechanism of homeostatic plasticity, then how does neuronal function recover? AIS geometry is unlikely to be the sole determinant of network operation after methylglyoxal exposure. For example, homeostatic increase of synaptic input could lead to recovery of network activity despite AIS shortening. Indeed, methylglyoxal increases both evoked and spontaneous EPSPs in cockroach abdominal ganglia (Chambers et al., 1985), but whether it alters synaptic efficacy in mouse cortical neurons is unknown. The *in vivo* situation could be similar. Type 2 diabetic db/db mice show AIS shortening (Yermakov et al., 2018, 2019), but there are also reports of altered synaptic plasticity (Li et al., 2002; Stranahan et al., 2008), decreased dendritic spine density (Stranahan et al., 2009; Chen et al., 2014), and progressive cortical atrophy (Ramos-Rodriguez et al., 2013). In addition, although not present in our *in vitro* model, elongation of the node of Ranvier after exposing myelinated CNS nerves to methylglyoxal *ex vivo* (Griggs et al., 2018) is similar to the elongated nodes present in older db/db mice (Yermakov et al., 2019). It is possible that the effects of methylglyoxal at the node of Ranvier *in vivo* could contribute to altered AIS geometry and network function. A better understanding of how concurrent changes at the AIS, node of Ranvier, and synapse affect network operation and cognitive function in neurological disease is needed. Finally, whether changes in AIS geometry and neuronal function are mechanistically linked remains an important unanswered question.

### AIS shortening may contribute to cognitive impairment

Current and previous results suggest that methylglyoxal-evoked AIS shortening could be involved in the pathophysiology of diabetic brain complications. Methylglyoxal metabolism is disrupted in patients with diabetes (Sveen et al., 2013; Andersen et al., 2018) or cognitive deficits (Beeri et al., 2011; Srikanth et al., 2013; Cai et al., 2014). In animals, a single intracerebroventricular administration (Qi et al., 2017; Lissner et al., 2021) or repeated injection (Hansen et al., 2016; Szczepanik et al., 2020) of methylglyoxal induces cognitive impairment. These observations suggest that persistent elevation of methylglyoxal, such as in diabetes, could produce long-lasting changes in the operation of CNS networks *in vivo.* Importantly, methylglyoxal is increased in db/db mice (Bierhaus et al., 2012; Griggs et al., 2019) with cognitive impairment and shortened AIS in the prefrontal cortex and hippocampus (Yermakov et al., 2018, 2019), suggesting a connection between methylglyoxal, type 2 diabetes, AIS shortening, and cognitive impairment. Strengthening a mechanistic link between subtle changes in AIS geometry and CNS dysfunction, pharmacological treatment of nerve-injured mice simultaneously alleviated both AIS shortening and cognitive impairment (Shiers et al., 2018).

In conclusion, our results indicate that methylglyoxal is a key mediator of AIS shortening, depolarization, and network activity changes in cultured cortical neurons. We speculate that the persistent alteration of neuronal function and AIS length by chronic disruption of methylglyoxal metabolism in neurological disease might contribute to cognitive impairment. Further investigation of the mechanisms of methylglyoxal-evoked AIS shortening and perturbation of neuronal operation could have a beneficial impact on a wide variety of conditions associated with disrupted methylglyoxal metabolism such as type 2 diabetes, Alzheimer’s disease, and aging.

## Synthesis

Reviewing Editor: Matthew Grubb, King’s College London Decisions are customarily a result of the Reviewing Editor and the peer reviewers coming together and discussing their recommendations until a consensus is reached. When revisions are invited, a fact-based synthesis statement explaining their decision and outlining what is needed to prepare a revision will be listed below. The following reviewer(s) agreed to reveal their identity: Paul Jenkins, Winnie Wefelmeyer. Note: If this manuscript was transferred from JNeurosci and a decision was made to accept the manuscript without peer review, a brief statement to this effect will instead be what is listed below.

Both reviewers note the potential for this manuscript to show that elevated methlyglyoxal can lead to alterations in AIS length. However, they raise a number of concerns that need to be addressed before the manuscript is suitable for publication in eNeuro. After consultation with the reviewers and myself, we would like to invite you to address all of the reviewers’ points below, with the following points of clarification:

- the methylglyoxal + TTX experiment suggested by Reviewer 2 (their point #1) needs to be carried out and incorporated into a revised version of the manuscript in order to clarify the proposed links between altered methylglyoxal and spiking activity

- AIS data need to be re-analysed on a culture-by-culture basis or, better, using multilevel/nested stats that take between-culture variability into account. Note that we *do not* require the live AIS label experiments suggested by Reviewer 1 (their point #2), though these would of course be very welcome.

- the manuscript must explicitly address the possibility that 100uM methylglyoxal is significantly beyond (patho)physiological levels in vivo (Reviewer 1 #1)

- the text throughout needs to be overhauled to avoid overinterpretation of the results (Reviewer 2, multiple points)

Reviewer 1:

This manuscript “The type 2 diabetes factor methylglyoxal mediates axon initial segment

shortening and neuronal network activity changes” attempts to relate increased levels of the glucose metabolite methylglyoxal with alterations in axon initial segment (AIS) morphology. The AIS is altered in diabetic mice, however the underlying mechanisms remain unclear. The authors propose that increased methylglyoxal levels, owing to chronic elevation of glucose, underlie these structural changes. Generally, the image quality is suitable and the manuscript is well-written and easy to read. However, several major flaws listed below preclude publication in its current form.

Major:

1. The authors cite a number of studies that have measured methylglyoxal levels, with those values ranging from 1-10 micromolar. They also cite one manuscript that they claim indicates 40 micromolar concentration, which serves as the rationale for studying a dose-response up to 100 micromolar. Liu at al. actually isolated dorsal root ganglion tissue and determined the amount of methylglyoxal (in nanomoles) as a function of grams of wet tissue weight. Their finding was 40 nmoles/g, not 40 micromolar. This is a potentially major issue since, excluding that study, the physiological range of methylglyoxal is 1-10 micromolar and in this study they use 100 micromolar for most of the experiments. This calls into question the rationale for this study and the validity of the conclusions.

2. The changes in AIS length are modest and it is unclear if the statistical significance is biologically relevant. The authors are including each AIS as an N, which is probably significantly oversampling with Ns in the 300-400 range. In addition, this data visualization style hides many of the points. The authors should use a scatter plot or some other means to display the data. Have the authors done power calculations to determine the sample size necessary to detect differences? How do the data look if each culture is averaged, instead of each individual AIS? Given the variability in AIS length in culture, these experiments might be more convincing with live labeling of the AIS and before and after measurements of the same cell (using physiological methylglyoxal concentrations).

Minor:

1. Fig 2 p value doesn’t match between figure legend and results.

2. The N values for control and methylglyoxal in Figure 7 should match. In some cases, the magnitude of changes in controls is similar or greater than in methylglyoxal, but the smaller N may preclude reaching significance.

Reviewer 2:

In their study, Griggs et al. reveal for the first time that methylglyoxal, a by-product of glycolysis, can shorten the AIS in cortical mouse cultures. At the same time, methylglyoxal changes network activity. These results are interesting as they add to our understanding of structural changes at the AIS and might lead to a better understanding of cognitive deficits observed in type 2 diabetes patients.

The authors first explore the effect of incubating cultures with varying levels of insulin, glucose and methylglyoxal on AIS length. While glucose had a significant but small impact on AIS length, methylglyoxal led to an average reduction of AIS length by 11.5%. It is interesting to note that this occurred in both excitatory and inhibitory neurons. Since the AIS is the site of action potential generation and AIS shortening has been shown to change neuronal excitability, the authors then probe network activity levels in the culture by using multi-electrode arrays. They observe that the activity briefly increases immediately after the addition of methylglyoxal for 3 minutes, before reducing, returning to baseline and then slightly reducing again.

It is not within the scope of the experiments performed in this study to determine whether it’s the changes in network activity that cause an AIS shortening or whether it is a direct result of the methylglyoxal. Neither is it within the scope of the results presented to determine whether the AIS shortening causes the change in network activity. Despite this, a large portion of the discussion is centred around these questions.

In relation to this point, the authors should address the following, specific comments:

1)

What is causing the change in AIS length? Increasing methylglyoxal levels for 24 hours shortens the AIS but also has an effect on network activity. Is the shortening therefore an activity-dependent mechanism or does methylglyoxal trigger an intracellular signalling cascade that leads to a change in AIS structure, independent of neuronal activity? I suggest the authors repeat the experiment of increasing methylglyoxal levels but this time add TTX at the same time to prevent network firing. TTX is not thought to change AIS length in culture (Grubb and Burrone 2010), therefore it would be informative to see whether methylglyoxal can still change the AIS length in the absence of network activity. It’s important to note that a membrane depolarisation can still lead to a change in AIS length in the absence of spiking activity (Grubb and Burrone 2010), but it appears that the membrane depolarisation caused by methylglyoxal should be relatively brief (<5 minutes; de Arriba et al. 2006).

2)

The immediate, but brief (3 minutes) increase in activity following addition of methylglyoxal is unlikely to lead to a change in AIS structure, at least according to our current understanding of AIS plasticity. The authors cite two studies that have observed relatively fast decreases in AIS length (Evans et al. 2015, Jamann et al. 2021), yet these studies increased KCl concentration for 1-3 hours. Section 3.6 of the manuscript uses these two studies as references to suggest that this brief increase in activity of 3 minutes might be causing the AIS shortening, without pointing out these important differences in timing. This has to be worded more carefully and the limitations of the results need to be acknowledged better.

3)

Similarly, the abstract speaks of a ’transient’ increase in activity, followed by a reduction from baseline at 3 hours. Since ’transient’ is not further quantified, this statement appears to suggest the increase in activity lasts for up to three hours, which would change interpretation of the results quite dramatically. This should be rephrased to reflect the changes in activity more correctly and avoid misleading those who only read the abstract.

4)

While it would be interesting to understand whether it is indeed the shortening of the AIS that leads to a return to baseline activity, it is interesting to note that baseline levels of activity are also present before AIS shortening, at 15 mins to 3hrs. Perhaps the limited number of time points looking at network activity after addition of methylglyoxal obscures a fluctuation of activity levels that is independent of AIS length. Indeed, the authors acknowledge themselves that AIS shortening does not always lead to a change in excitability. Establishing actual causation here will be difficult and is clearly outside the scope of this study. In light of this and the relatively modest decreases in activity levels, I urge the authors to be careful and not suggest causation, such as in the abstract (“development of AIS shortening at 24h was associated with restoration of spiking to baseline levels”), and propose to simply reduce the focus on the functional impact of AIS plasticity (particularly in the discussion).

<b>Minor, other points</b>

Results paragraph 3.1 doesn’t describe the insulin protocol correctly, as insulin concentration was in fact changed for 48 hours from the default, high insulin state, first to 0 mM for 24h and then to a varying number for the last 24 hours. This was quite confusing at first, it’s worthwhile clarifying this in the text.

How much glucose is in culture medium normally? Was it increased to 25 mM or by 25 mM?

Figure 2: please include example images of AIS before and after high glucose.

The authors state in the text that methylglyoxal did not change the AIS start position, however this data is not shown. It would be good to include this in figure 4.

Figure 5: figure legend states 5B quantifies cell viability results after 24 or 72 hours, whereas the graph only shows a comparison at 24 hours.

Figure 6: I could not find any mention of how many preparations this was done in, please could this be added in the results text or the figure legend.

## Author Response

Synthesis Statement for Author (Required):

Both reviewers note the potential for this manuscript to show that elevated methlyglyoxal can lead to alterations in AIS length. However, they raise a number of concerns that need to be addressed before the manuscript is suitable for publication in eNeuro. After consultation with the reviewers and myself, we would like to invite you to address all of the reviewers’ points below, with the following points of clarification:

- the methylglyoxal + TTX experiment suggested by Reviewer 2 (their point #1) needs to be carried out and incorporated into a revised version of the manuscript in order to clarify the proposed links between altered methylglyoxal and spiking activity

o The methylglyoxal+TTX experiment is now included in the revision as Figure 10. In addition, we go beyond this suggestion by including whole-cell patch clamp electrophysiology analysis of the effect of methylglyoxal on resting membrane potential and other parameters of single-cell excitability (Fig. 7) at the same timepoints that we measured AIS length and MEA network activity (0.5, 3, 24 h).

- AIS data need to be re-analysed on a culture-by-culture basis or, better, using multilevel/nested stats that take between-culture variability into account. Note that we *do not* require the live AIS label experiments suggested by Reviewer 1 (their point #2), though these would of course be very welcome.

o We reanalyzed AIS data on a culture-by-culture basis and present the data at both the AIS and culture level in the revised Figures and Results text.

- the manuscript must explicitly address the possibility that 100uM methylglyoxal is significantly beyond (patho)physiological levels in vivo (Reviewer 1 #1)

o We now provide a more detailed rationale for our chosen concentration of methylglyoxal and added text that explicitly addresses the possibility that 100 μM methylglyoxal in our cortical culture model may be beyond pathophysiological levels in vivo.

- the text throughout needs to be overhauled to avoid overinterpretation of the results (Reviewer 2, multiple points)

o We substantially revised the text to 1) avoid overinterpretation of our original results and 2) to incorporate discussion of the new results (new Figs. 7,10).

Reviewer 1:

This manuscript “The type 2 diabetes factor methylglyoxal mediates axon initial segment

shortening and neuronal network activity changes” attempts to relate increased levels of the glucose metabolite methylglyoxal with alterations in axon initial segment (AIS) morphology. The AIS is altered in diabetic mice, however the underlying mechanisms remain unclear. The authors propose that increased methylglyoxal levels, owing to chronic elevation of glucose, underlie these structural changes. Generally, the image quality is suitable and the manuscript is well-written and easy to read. However, several major flaws listed below preclude publication in its current form.

Major:

1. The authors cite a number of studies that have measured methylglyoxal levels, with those values ranging from 1-10 micromolar. They also cite one manuscript that they claim indicates 40 micromolar concentration, which serves as the rationale for studying a dose-response up to 100 micromolar. Liu at al. actually isolated dorsal root ganglion tissue and determined the amount of methylglyoxal (in nanomoles) as a function of grams of wet tissue weight. Their finding was 40 nmoles/g, not 40 micromolar. This is a potentially major issue since, excluding that study, the physiological range of methylglyoxal is 1-10 micromolar and in this study they use 100 micromolar for most of the experiments. This calls into question the rationale for this study and the validity of the conclusions.

We agree that the concentrations of methylglyoxal used in our in vitro study are an important consideration. The PATHOphysiological levels of methylglyoxal within CNS tissues in vivo are not established, although there is some evidence of increase of methylglyoxal in the blood in neurological conditions. Even the information available in the literature about normal, physiological levels of methylglyoxal in CNS tissue is limited and is somewhat controversial, since some methods used to measure methylglyoxal may be inaccurate due to inappropriate sample processing (see (Rabbani and Thornalley, 2014) for detail). Of note, we did not consider reports of methylglyoxal levels that used inappropriate methods. Since the normal levels of methylglyoxal in vitro are not known, we used the previous literature of in vivo methylglyoxal levels in CNS tissue (1-40 μM) as a guide. We empirically determined the concentration required to disrupt cellular methylglyoxal metabolism in vitro (Fig. 3) by exposing our cortical cultures to 1 μM (probably close to normal), 10 μM (perhaps normal or perhaps elevated), and 100 μM (definitely elevated) methylglyoxal. We found that 100 μM but not 10 μM or 1 μM methylglyoxal exposure for 24 h was sufficient to disrupt cellular methylglyoxal metabolism in our cortical cultures, as evidenced by an increase in methylglyoxal-H1 AGE (Fig. 3). We also found that 100 μM but not 10 μM or 1 μM methylglyoxal shortened the AIS at the culture level (Fig. 4) and was non-toxic (Fig. 5). These methylglyoxal-H1 AGE, AIS geometry, and cell viability data suggest that adding 100 μM methylglyoxal to our cortical cultures could be a pathophysiologically-relevant in vitro model.

In the revision, we now discuss some of the studies that might have overestimated methylglyoxal levels in the brain and note that the levels of methylglyoxal in CNS tissue are not well-established, especially during PATHOphysiological conditions. We also note in the revision how we, for ease of reading, converted 40 nmol/g in Liu et al 2017 to micromolar concentration assuming a density of isolated dorsal root ganglia tissue of 1 g/mL (40 nmol/g x 1 g/mL x 1000 mL/L = 40 umol/L). We think it reasonable to include Liu et al 2017 when considering the physiological, normal range of methylglyoxal concentration to be 1-40 micromolar. Even without this study we validated that 100 μM but not 10 μM methylglyoxal was required to disrupt cellular methylglyoxal metabolism (Fig. 3), suggesting that 10 μM may be physiological whereas 100 μM may be pathophysiological in our culture model.

To better reflect this complicated situation, we revised Results section 3.3 as follows:

"To test the hypothesis that accumulation of methylglyoxal disrupts AIS geometry, we first needed to determine the concentration of methylglyoxal exposure required to disrupt cellular methylglyoxal metabolism in our in vitro cortical culture model. Reliable estimates of methylglyoxal concentration in the in vivo nervous system during normal, physiological conditions are 1.2 μM (Eberhardt et al., 2012) or 40 nmol/g (Liu et al., 2017) in control dorsal root ganglia, 0.3-1.5 μM (Kurz et al., 2011) or 5 μM (Distler et al., 2012) in young adult mouse brain, and 10 μM in the cerebrospinal fluid of healthy control middle-aged humans (Kuhla et al., 2005). Conversion of 40 nmol/g in Liu et al (2017) to a micromolar concentration assuming a density of DRG tissue of ∼1 g/mL yields 40 μM methylglyoxal. Thus, these studies suggest the normal concentration of methylglyoxal in the brain could be 1-40 μM. Other studies report methylglyoxal levels in whole brain as high as 170-360 μM (Hambsch et al., 2010) but this may be an overestimation due to inappropriate sample processing (Rabbani and Thornalley, 2014). Information about methylglyoxal levels in CNS tissue during pathophysiological disease conditions such as type 2 diabetes are not well-established. Increased methylglyoxal concentration in the blood or serum of patients with type 2 diabetes (Andersen et al., 2018), Alzheimer’s disease (Haddad et al., 2019), and aging-related cognitive decline (Beeri et al., 2011) suggest that levels of methylglyoxal may increase in CNS tissues during neurological disease. To validate the concentration of methylglyoxal required to produce pathophysiological disruption of cellular methylglyoxal metabolism in our postnatal cortical cultures, we tested if 24 h exposure to 0, 1, 10, or 100 μM methylglyoxal would increase hydroimidazolone (methylglyoxal-H1), a methylglyoxal-derived advanced glycation end-product (methylglyoxal-AGE). methylglyoxal-H1 is a suitable marker for disrupted cellular methylglyoxal metabolism since pathophysiological increase of methylglyoxal leads to accumulation of methylglyoxal-AGEs in vivo, and upwards of 90% of methylglyoxal-AGEs in vivo are the methylglyoxal-H1 moiety (Rabbani and Thornalley, 2012). methylglyoxal-H1 and Total Protein in immunoblots of postnatal cortical culture cell lysates are shown (Fig. 3A). methylglyoxal-H1 relative band density (normalized to total protein lane volume) was increased by 100 μM methylglyoxal (p = 0.001) but not 1 or 10 μM (F (3, 8) = 16.08; p = 0.0009; Dunnett; n=3 cultures per dose) (Fig. 3B). These results indicate that adding 100 μM methylglyoxal for 24 h is sufficient to acutely disrupt cellular methylglyoxal metabolism in cortical cultures, suggesting that this is a pathophysiologically-relevant dose in our in vitro model.”

We modified the intro for Results section 3.4 as follows:

"To test if methylglyoxal shortens the AIS, we exposed mature neuronal networks to a range of concentrations (0-100 μM) in vitro that likely represent physiological to potentially pathophysiological levels of methylglyoxal within in vivo CNS tissue for 24 h.”

We modified the Discussion section 4.1 to explicitly acknowledge the possibility that 100 μM methylglyoxal in vitro may not be pathophysiologically-relevant in the in vivo condition:

"However, we recognize the reductionist in vitro approach herein cannot completely recapitulate chronic diabetic conditions in vivo. We based our study on the limited information available in the literature suggesting 1-40 μM is the normal, physiological range of methylglyoxal in CNS tissues (Kuhla et al., 2005; Kurz et al., 2011; Distler et al., 2012; Eberhardt et al., 2012; Rabbani and Thornalley, 2014; Liu et al., 2017). Since cellular levels of methylglyoxal in the brain during neurological disease are not established, it is possible that the 100 μM methylglyoxal required to increase cellular methylglyoxal-H1 and produce AIS shortening in the current in vitro study does not represent pathophysiological levels in vivo. Nevertheless, it is reasonable to think that prolonged increase of methylglyoxal, or the combined disruption of methylglyoxal, insulin, and glucose metabolism, during type 2 diabetes could result in sustained AIS shortening and CNS dysfunction.”

Lastly, we added the word “cultured” to our main conclusion in Discussion section 4.7 and added a speculative statement about how our results might pertain to the in vivo situation:

"Our results indicate that methylglyoxal is a key mediator of AIS shortening, depolarization, and network activity changes in cultured cortical neurons. We speculate that the persistent alteration of neuronal function and AIS geometry by chronic disruption of methylglyoxal metabolism in neurological disease might contribute to cognitive impairment.”

2. The changes in AIS length are modest and it is unclear if the statistical significance is biologically relevant. The authors are including each AIS as an N, which is probably significantly oversampling with Ns in the 300-400 range. In addition, this data visualization style hides many of the points. The authors should use a scatter plot or some other means to display the data. Have the authors done power calculations to determine the sample size necessary to detect differences? How do the data look if each culture is averaged, instead of each individual AIS? Given the variability in AIS length in culture, these experiments might be more convincing with live labeling of the AIS and before and after measurements of the same cell (using physiological methylglyoxal concentrations).

The biological relevance of the 11.5% AIS shortening produced by methylglyoxal is highlighted in the revised Discussion section 4.1:

"In contrast, increase of methylglyoxal (100 μM) produced 11.5% AIS shortening (Fig. 4) that was recoverable and not associated with cell death (Fig. 5). This is consistent with 8-16% AIS shortening in the prefrontal cortex of type 2 diabetic db/db mice at 10 weeks of age with lack of apoptotic cell death (Yermakov et al., 2018). Methylglyoxal elevation leads to accumulation of methylglyoxal-H1 in patients with type 2 diabetes (Schalkwijk and Stehouwer, 2020) and both methylglyoxal (Bierhaus et al., 2012) and methylglyoxal-H1 (Griggs et al., 2019) are elevated in db/db mice that also display AIS shortening and cognitive impairment (Yermakov et al., 2018, 2019).”

The biological relevance of modest AIS length changes in general is highlighted in the revised Results section 3.7:

"Subtle AIS shortening (a change in length as small as 4.5%) is reported to reduce neuronal excitability at the cellular (Baalman et al., 2013; Evans et al., 2015; Vascak et al., 2017; Goethals and Brette, 2020; Jamann et al., 2021) and network (Fruscione et al., 2018; Sohn et al., 2019) levels. In the next experiments we tested the hypothesis that methylglyoxal-evoked AIS shortening would be associated with reduced neuronal function at both the cellular and network levels.”

As requested by the Reviewer and Editor we re-analyzed the results on a culture-by-culture basis (n = average of each culture preparation). We added several culture preparations to the time course analysis of AIS length after methylglyoxal exposure (revised Fig. 11) to enable analysis on a culture-by-culture basis, since the data in the original submission (original Fig. 7) was from 1 culture preparation. The reduction of AIS length by 100 μM methylglyoxal is statistically significant when analyzed at the AIS, culture, or nested level (nested level is not shown in the revision but we did check this type of analysis for Figure 4 methylglyoxal dose response). Almost all other data look similar whether analyzed at the AIS or culture level, except the minor shortening in AIS length produced by 50 mM glucose is no longer statistically significant when analyzed at the culture level. This strengthens the conclusion that methylglyoxal is the key diabetes-related factor that mediates AIS shortening within our in vitro cortical cultures.

To reflect the analysis at both AIS and culture-by-culture basis we made the following changes to the manuscript. We updated the Results text to reflect the statistics at both the AIS and culture level. We changed the data visualization style of the Figures to show both each AIS (small grey dots) and the average values at the culture preparation level (larger open symbols). We modified Methods section 2.9 as follows:

"Statistics for both n=AIS/image/coverslip and n=culture preparation (average of AIS geometry, cell viability, or cell distribution outcomes within a single culture preparation) are reported in the results text. Comparison of the means between two groups was performed using unpaired, homoscedastic t-test. Effect of insulin, glucose, or methylglyoxal concentration was analyzed using one-way ANOVA and/or linear regression. An interaction of time and drug was determined using two-way ANOVA. Multiple comparison post-hoc tests are listed in the results text. An alpha value of α=0.05 was used to determine statistical significance. All data were analyzed using Prism 9.1 (GraphPad, La Jolla, CA) or SAS 9.4 (SAS Institute, Inc., Cary, NC). Data for n=AIS are presented as cumulative frequency distributions or scatter plots where the width of the distribution of data points is proportional to the number of data points at a given Y-value. Data for n=culture preparation (larger open symbols) are overlayed on the scatter plots showing each AIS, image, or coverslip (small grey dots). Figures show mean {plus minus} SEM and the p-value or “ns” (not significant) for n=culture preparation (immunofluorescence) or n=MEA/cell (electrophysiology).”

Minor:

1. Fig 2 p value doesn’t match between figure legend and results.

This is no longer an issue since we now present p-values for statistics on a culture-by-culture basis. The new p-values in Figure 2 are listed as “ns” since they are not significant at the culture level. The originally reported p-value of p = 0.04 for the Tukey correction for multiple comparisons of 25 mM glucose versus 50 mM glucose was correct.

2. The N values for control and methylglyoxal in Figure 7 should match. In some cases, the magnitude of changes in controls is similar or greater than in methylglyoxal, but the smaller N may preclude reaching significance.

It is unclear in which cases the reviewer expects to reach (statistical) significance in control MEA outcomes in Figure 7 of the original manuscript (revised Fig. 9). We do not think adding n=3 control MEA at this stage of our study is appropriate nor would it change the conclusions of the study.

Reviewer 2:

In their study, Griggs et al. reveal for the first time that methylglyoxal, a by-product of glycolysis, can shorten the AIS in cortical mouse cultures. At the same time, methylglyoxal changes network activity. These results are interesting as they add to our understanding of structural changes at the AIS and might lead to a better understanding of cognitive deficits observed in type 2 diabetes patients.

The authors first explore the effect of incubating cultures with varying levels of insulin, glucose and methylglyoxal on AIS length. While glucose had a significant but small impact on AIS length, methylglyoxal led to an average reduction of AIS length by 11.5%. It is interesting to note that this occurred in both excitatory and inhibitory neurons. Since the AIS is the site of action potential generation and AIS shortening has been shown to change neuronal excitability, the authors then probe network activity levels in the culture by using multi-electrode arrays. They observe that the activity briefly increases immediately after the addition of methylglyoxal for 3 minutes, before reducing, returning to baseline and then slightly reducing again.

It is not within the scope of the experiments performed in this study to determine whether it’s the changes in network activity that cause an AIS shortening or whether it is a direct result of the methylglyoxal. Neither is it within the scope of the results presented to determine whether the AIS shortening causes the change in network activity. Despite this, a large portion of the discussion is centred around these questions.

In relation to this point, the authors should address the following, specific comments:

[Major:]

1) What is causing the change in AIS length? Increasing methylglyoxal levels for 24 hours shortens the AIS but also has an effect on network activity. Is the shortening therefore an activity-dependent mechanism or does methylglyoxal trigger an intracellular signalling cascade that leads to a change in AIS structure, independent of neuronal activity? I suggest the authors repeat the experiment of increasing methylglyoxal levels but this time add TTX at the same time to prevent network firing. TTX is not thought to change AIS length in culture (Grubb and Burrone 2010), therefore it would be informative to see whether methylglyoxal can still change the AIS length in the absence of network activity. It’s important to note that a membrane depolarisation can still lead to a change in AIS length in the absence of spiking activity (Grubb and Burrone 2010), but it appears that the membrane depolarisation caused by methylglyoxal should be relatively brief (<5 minutes; de Arriba et al. 2006).

We performed the suggested TTX+methylglyoxal experiment. We now show that 100 μM methylglyoxal produces AIS shortening regardless of whether spiking is blocked by co-incubation with 1 μM TTX (Fig. 10). In addition, our results indicate that TTX alone (and thus spike blockade) does not change AIS length in our cortical cultures. These new results suggest that the change in AIS length we observed after adding methylglyoxal is independent of its effect on neuronal spiking activity. Please see the revised Results section 3.9.

We also assessed the time course of changes in resting membrane potential after methylglyoxal exposure (revised Fig. 7). These results show that the membrane depolarization induced by methylglyoxal in our cortical cultures occurs within minutes, persists for 0.5-3 h, and then returns to normal at 24 h. Please see the revised Results section 3.7.

We moved the time course analysis of methylglyoxal-evoked AIS shortening to revised Figure 11. Please see the revised Results section 3.10.

Taken together, these results indicate that although methylglyoxal depolarizes resting membrane potential and reduces MEA network activity at 0.5-3 h, this is not associated with rapid AIS shortening since AIS length is similar at 3 h after methylglyoxal exposure. Instead, AIS shortening at 24 h is associated with recovery of both resting membrane potential and network activity. Furthermore, the new TTX+methylglyoxal results are consistent with the idea proposed by others (see (Grubb and Burrone, 2010; Evans et al., 2015)) that AIS plasticity is independent of network spiking activity. We agree that our experiments do not identify the signaling cascade(s) involved, but we do provide some plausible mechanisms of methylglyoxal-evoked AIS shortening in the Discussion section 4.2.

Based on the comments from the Editor and Reviewer 2, we substantially overhauled the manuscript to avoid overinterpretation and to incorporate these new results (Fig. 7: methylglyoxal depolarization; Fig. 10: TTX+methylglyoxal) into a better Discussion. Please see the revised Discussion sections 4.2, 4.3, 4.4.

2) The immediate, but brief (3 minutes) increase in activity following addition of methylglyoxal is unlikely to lead to a change in AIS structure, at least according to our current understanding of AIS plasticity. The authors cite two studies that have observed relatively fast decreases in AIS length (Evans et al. 2015, Jamann et al. 2021), yet these studies increased KCl concentration for 1-3 hours. Section 3.6 of the manuscript uses these two studies as references to suggest that this brief increase in activity of 3 minutes might be causing the AIS shortening, without pointing out these important differences in timing. This has to be worded more carefully and the limitations of the results need to be acknowledged better.

We agree this was confusing based on the differences in timing and the very transient (∼1min) increase of activity by methylglyoxal. We no longer think that this brief increase in activity might be the cause of AIS shortening since we now incorporate new data showing that methylglyoxal-evoked AIS shortening is independent of neuronal spiking (revised Fig. 10: TTX+methylglyoxal) and that methylglyoxal rapidly depolarizes resting membrane potential (revised Fig. 7).

To word this more carefully, we removed the following statements from the revised Results section 3.8:

"Burgeoning research suggests activity-dependent structural plasticity of AIS geometry is a mechanism of fine-tuning neuronal excitability (Yamada and Kuba, 2016; Jamann et al., 2018). For example, depolarization by high KCl induces AIS shortening at 1 h (Jamann et al., 2021), 3 h (Evans et al., 2015), or 24 h (Chand et al., 2015).”

"Acute increase of network activity by methylglyoxal could lead to rapid (within 1-3 h) activity-dependent AIS shortening, so in the next experiments we characterized the time course of AIS shortening and network activity changes.”

We also combined the rationale for assessing the changes in MEA network activity at both ’immediate’ and ’delayed’ time points in the revised Results section 3.8 as follows:

"Our results (Fig. 7) and previous studies indicate that methylglyoxal acutely alters neuronal activity at the cellular level (de Arriba et al., 2006; Bierhaus et al., 2012; Radu et al., 2012; Andersson et al., 2013; Griggs et al., 2019). To determine the effect of methylglyoxal on the coordinated activity of neuronal networks we utilized multi-electrode array (MEA) recordings of our cortical cultures.”

"We also recorded from the same MEAs at later timepoints to examine how cellular depolarization at 0.5-3 h or AIS shortening at 24 h affect neuronal network activity.”

3) Similarly, the abstract speaks of a ’transient’ increase in activity, followed by a reduction from baseline at 3 hours. Since ’transient’ is not further quantified, this statement appears to suggest the increase in activity lasts for up to three hours, which would change interpretation of the results quite dramatically. This should be rephrased to reflect the changes in activity more correctly and avoid misleading those who only read the abstract.

We rephrased the abstract to reflect the changes in cellular and network activity more correctly as follows:

"In single-cell recordings resting membrane potential was depolarized at 0.5-3 h and returned to normal at 24 h. In multi-electrode array recordings methylglyoxal produced an immediate ∼300% increase in spiking and bursting rates that returned to normal within 2 min, followed by a ∼20% reduction of network activity at 0.5-3 h and restoration of activity to baseline levels at 24 h.”

4) While it would be interesting to understand whether it is indeed the shortening of the AIS that leads to a return to baseline activity, it is interesting to note that baseline levels of activity are also present before AIS shortening, at 15 mins to 3hrs. Perhaps the limited number of time points looking at network activity after addition of methylglyoxal obscures a fluctuation of activity levels that is independent of AIS length. Indeed, the authors acknowledge themselves that AIS shortening does not always lead to a change in excitability. Establishing actual causation here will be difficult and is clearly outside the scope of this study. In light of this and the relatively modest decreases in activity levels, I urge the authors to be careful and not suggest causation, such as in the abstract (“development of AIS shortening at 24h was associated with restoration of spiking to baseline levels”), and propose to simply reduce the focus on the functional impact of AIS plasticity (particularly in the discussion).

We agree that establishing causation between AIS shortening and changes in cellular and network function is difficult. We substantially overhauled the text to reflect the implications of the new results (Fig. 7: methylglyoxal depolarization; Fig. 10: TTX+methylglyoxal) and to reduce the focus on the functional impact of AIS plasticity, particularly in the discussion. For example, we removed the following statements (originally in Discussion section 4.2) from the revised Discussion:

"Little is known about the influence of AIS shortening on neuronal network operation and thus cognitive function.”

"At the network level, one study reported AIS shortening and reduced MEA spiking, but the cultures consisted of only excitatory neurons (Sohn et al., 2019). Here, we measured AIS geometry and MEA activity in neuronal networks containing both excitatory and inhibitory neurons. We observed AIS shortening in both CaMKII+ and CaMKII- neurons (Fig. 8) when network spiking returned to basal levels at 24 h, an increase from diminished activity at 3 h (Fig. 7). This seems counterintuitive if the current understanding is that AIS shortening reduces excitability. However, comparison of single neuron excitability versus MEA activity is difficult since many factors (e.g., composition of the neuronal network) can dictate network operation. We offer some speculative explanations. First, if inhibitory output plays a predominant role in network activity, then shortened AIS in inhibitory neurons would reduce the inhibitory drive onto excitatory neurons causing network activity to increase. This suggests diminished network activity when AIS shortening occurs in systems consisting of excitatory neurons only. Indeed, shorter AIS length associated with reduced MEA network burst rate in human iPSC-derived excitatory neuron cultures lacking inhibitory neurons (Sohn et al., 2019). Second, AIS shortening could reduce excitability in inhibitory neurons but not excitatory neurons since differences in the size or shape of these neurons can affect how AIS shortening modifies their excitability (Gulledge and Bravo, 2016). Third, altered Nav phosphorylation or Kv1 levels (discussed above) could differentially affect the functional outcome of AIS shortening in excitatory versus inhibitory neurons. Finally, spontaneous spiking does not reveal how underlying changes (i.e., AIS shortening) in the network might affect the response to subsequent perturbation. Thus, homeostatic capability of the network could still be impaired. This was previously reported for mutant tau excitatory neurons with shorter AIS, which led to a blunted response in MEA activity after challenge with depolarizing KCl (Sohn et al., 2019).”

We also removed the sentence quoted by Reviewer 2 (“development of AIS shortening at 24h was associated with restoration of spiking to baseline levels”) from the Abstract.

We did assess network activity at 0.5 h and 6 h but did not include these timepoints in the original submission. Even though network activity returns to baseline within 15 min, it is indeed reduced at 0.5 h, 3 h, and 6 h. Since we characterized both single-cell excitability and AIS length at 0.5-3 h, we added the 0.5 h timepoint for Network spike rate to the revised Figure 9. We did not add the 6 h timepoint since it is virtually identical to 3 h.

Even though our study cannot establish causation between AIS shortening and neuronal function, we thought it would be helpful to discuss how AIS shortening by methylglyoxal and the effects of methylglyoxal on cellular and network function might be linked. Thus, we created the new Discussion section 4.4 entitled: “Are AIS geometry and neuronal function linked?”. This section ends with the following statement:

"Finally, whether changes in AIS geometry and neuronal function are mechanistically linked remains an important unanswered question.”

Minor, other points:

Results paragraph 3.1 doesn’t describe the insulin protocol correctly, as insulin concentration was in fact changed for 48 hours from the default, high insulin state, first to 0 mM for 24h and then to a varying number for the last 24 hours. This was quite confusing at first, it’s worthwhile clarifying this in the text.

Thank you for noticing this discrepancy. The protocol is correctly described in the Methods text and in Figure 1A, but not in the Results 3.1 text. To correctly describe the insulin exposure protocol we revised the Results section 3.1 text as follows:

"We utilized a protocol previously established to produce insulin resistance after 24 h exposure to insulin in embryonic mouse primary cortical cultures (Kim et al., 2011). As shown in the experimental design (Fig. 1A), we removed insulin for 24 h in mature cultures, varied the insulin concentration for an additional 24 h, then validated the presence of insulin resistance via Akt immunoblotting and quantified AIS length by immunostaining for AnkryinG, the master organizer of the AIS (Rasband, 2010). Previous studies suggest that a 24 h period of time might be sufficient to observe changes in AIS geometry in cultured CNS neurons (Grubb and Burrone, 2010; Chand et al., 2015).”

How much glucose is in culture medium normally? Was it increased to 25 mM or by 25 mM?

Normal culture medium contains 25 mM glucose. For the high glucose condition, glucose was increased from 25 mM to 50 mM (so it was increased by 25 mM). To improve clarity in the revision, we made the following changes:

- modified Methods section 2.3 Glucose exposure. “To model hyperglycemia in mature (DIV 10-11) cultures, coverslips were grown in standard culture conditions from DIV 0-10 and then exposed to unmodified media containing 25 mM glucose or media modified to contain 50 mM high glucose or 25 mM normal glucose plus 25 mM mannitol.”

- modified Results section 3.2 as follows: “Example images are shown 24 h after exposing mature cortical cultures to normal Neurobasal-A Growth Media containing 25 mM glucose, high glucose media containing 50 mM glucose, or normal media containing 25 mM glucose with 25 mM mannitol added to control for the elevated osmolarity in high glucose media (Fig. 2A).”

Figure 2: please include example images of AIS before and after high glucose.

We added the example images as Figure 2A.

The authors state in the text that methylglyoxal did not change the AIS start position, however this data is not shown. It would be good to include this in figure 4.

We added the AIS start location data after methylglyoxal exposure (revised Fig. 4D-E). We also changed the term “position” to “location” in the revised manuscript to maintain consistency when referring to the definition of AIS geometry.

Figure 5: figure legend states 5B quantifies cell viability results after 24 or 72 hours, whereas the graph only shows a comparison at 24 hours.

Thank you for noticing this error. It has been corrected in the revision (removed “or 72 h”).

Figure 6: I could not find any mention of how many preparations this was done in, please could this be added in the results text or the figure legend.

We added the following statement to revised Figure 8B,C legend and to revised Results section 3.8:

"(n=6 MEAs from 2 preparations for media and n=9 MEAs from 3 preparations for methylglyoxal)"
